# Mortality trends in idiopathic pulmonary fibrosis in Europe between 2013 and 2018

**DOI:** 10.1183/13993003.02080-2023

**Published:** 2024-08-22

**Authors:** Francesca Gonnelli, Martina Bonifazi, Richard Hubbard

**Affiliations:** 1 Lifespan and Population Health, University of Nottingham, Nottingham, UK; 2 Respiratory Unit, Department of Biomedical Sciences and Public Health, Polytechnic University of Marche, Ancona, Italy; 3 Interstitial Lung Diseases, Pleural Diseases and Bronchiectasis Unit, Azienda Ospedaliero-Universitaria delle Marche, Ancona, Italy

## Abstract

**Background:**

Previous research has suggested that the incidence of idiopathic pulmonary fibrosis (IPF) is increasing in the UK and elsewhere. The aim of this study is to provide contemporary estimates of IPF mortality rates across 24 European Union (EU) countries from 2013 to 2018, using death certificate data from the European Statistics Institution (EUROSTAT) database.

**Methods:**

We extracted country data for IPF (International Classification of Diseases, 10th Revision: code J84.1) mortality from the EUROSTAT dataset. We calculated country-, age- and sex-specific death registration rates between 2013 and 2018. We used direct standardisation to compare rates between countries. We calculated annual trends in mortality rate ratios using a segmented regression model.

**Results:**

The overall standardised mortality rate in 24 EU countries during this period was 3.90 (95% CI 3.80–3.90) per 100 000 person-years, with the rate rising from 3.70 in 2013 to 4.00 in 2018 (average annual percent change 1.74%, 95% CI 0.91–2.59%). We observed substantial inter-country differences, with the highest rates detected in Ireland, the UK and Finland, the lowest rate in Bulgaria, and middle rates in Germany, Greece, Italy, the Netherlands, Portugal and Slovenia.

**Conclusions:**

The IPF mortality rate is increasing across Europe. There are currently more than 17 000 deaths recorded from IPF each year in Europe but the marked geographical differences we observed suggest that this figure may underestimate the true rate considerably.

## Extract

Previous studies in the UK [1] and Europe [2] have shown that the mortality from idiopathic pulmonary fibrosis (IPF) has increased over time, but up-to-date studies using consistent methodology are limited. The last reliable European estimate of IPF mortality was provided by Marshall *et al*. [2] using World Health Organization (WHO) data. Their study showed that the mortality rate from IPF increased in the 17 European countries studied from 2001 to 2013, with substantial geographical variations.

## Shareable abstract

**The mortality rate of idiopathic pulmonary fibrosis is on the rise across Europe. Considerable differences exist among countries. Currently, more than 17 000 deaths from IPF are recorded each year in Europe, with a high probability of underestimation.** https://bit.ly/3yLyujQ

## Graphical abstract

**Figure:**
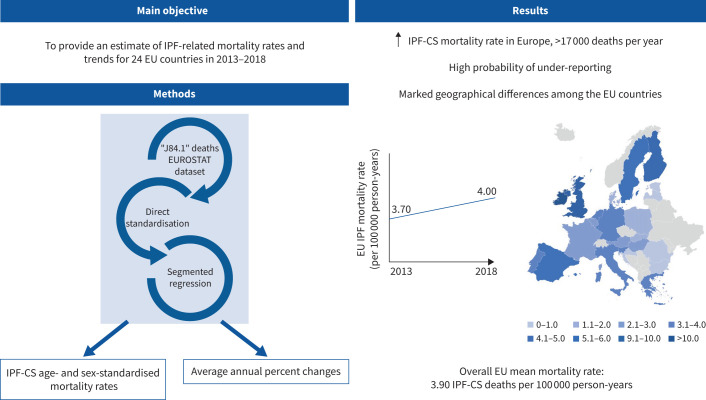
Overview of the study. IPF: idiopathic pulmonary fibrosis; IPF-CS: idiopathic pulmonary fibrosis-clinical syndrome; EU: European Union.

## Introduction

Previous studies in the UK [1] and Europe [2] have shown that the mortality from idiopathic pulmonary fibrosis (IPF) has increased over time, but up-to-date studies using consistent methodology are limited. The last reliable European estimate of IPF mortality was provided by Marshall *et al*. [2] using World Health Organization (WHO) data. Their study showed that the mortality rate from IPF increased in the 17 European countries studied from 2001 to 2013, with substantial geographical variations.

The primary objective of our study is to provide a contemporary estimate of IPF-related mortality rates, trends and variations for 24 European Union (EU) countries from 2013 to 2018, using the European Statistics Institution (EUROSTAT) dataset [3].

In addition, we correlated IPF-clinical syndrome (IPF-CS) mortality data with interstitial lung disease (ILD) (including sarcoidosis) prevalence rates in the same countries and the same years, using the Global Burden of Disease (GBD) dataset.

## Methods

### IPF definition

Since 2011 all European countries are obliged to provide yearly disease-specific mortality data using the International Classification of Diseases, 10th Revision (ICD-10) coding system. For our study we defined death from IPF as deaths coded as ICD-10 J84.1 [4]. In addition, we conducted a second analysis using a broader definition for IPF through all the J84 codes. Since our data represent death certification diagnoses we have used the term IPF-CS throughout the text, which is consistent with our other studies [1, 5–7].

### Dataset

#### EUROSTAT dataset: mortality study

The EUROSTAT dataset contains principal cause of mortality data derived from death certificates. This dataset is publicly available online from the website [3] and additional data can be gained through the “users support” platform. We extracted deaths from ICD-10 J84 and J84.1, and additional disease demographic data (overall European population, stratified by sex, age and country). We identified deaths in 24 countries (*i.e.* Austria, Belgium, Bulgaria, Croatia, Denmark, Estonia, Finland, France, Germany, Greece, Hungary, Ireland, Italy, Luxembourg, Latvia, the Netherlands, Poland, Portugal, Romania, Slovenia, Slovakia, Spain, Sweden and the UK) in the EU between 2013 and 2018, stratified by age and sex. We did not include data from 2019 because data for Greece, France and the UK were missing for this year. We grouped data on registered deaths and general population data into 5-year age bands between ages 55 and 85 years. We used total resident populations for each year and for each country as our denominators.

A sample of the dataset is included in supplementary material S1.

#### GBD dataset: disease prevalence study

Information on disease prevalence for each specific ILD is not available in the GBD, but we were able to extract data on the prevalence of overall ILD including sarcoidosis. We extracted these data for the years 2013–2018 for all 24 of the EU countries. Data were provided directly as prevalence rates. More details about the GBD dataset are reported in supplementary material S2.

### Statistical analysis

We used Stata version 18 (StataCorp, College Sation, TX, USA) for data management and statistical analysis, and the Surveillance Epidemiology and End Results statistical software (Joinpoint Regression Program version 5; https://surveillance.cancer.gov/joinpoint) for the segmented regression to obtain the mortality average annual percent change (AAPC), and trends and their statistical significance (supplementary material S3).

We wrote the manuscript in accordance with the Reporting of studies Conducted using Observational Routinely-collected health Data (RECORD) statement [7].

#### Mortality study

For each country, we calculated annual crude mortality rates stratified by sex and age group. We estimated annual standardised mortality rates for each country, using direct standardisation with the European population in 2017 as our reference population (supplementary material S4). More details about the direct standardisation technique are provided in supplementary material S5.

To obtain a more reliable snapshot of the IPF mortality distribution across Europe, we focused on the central years of the study period, excluding years with missing data (*i.e.* 2013 and 2018). We calculated overall age- and sex-adjusted mortality rates for Europe and each European country from 2015 to 2017. We displayed the results both through a two-way scatter plot and a geographical map.

We used a segmented regression model to analyse mortality rate trends over time for each country and for all Europe, overall and stratified by sex and age.

The geographical distribution of rate ratios was visualised and summarised using a map graph and a two-way scatter plot.

We then repeated our analyses using the broader definition based on J84 codes.

#### Prevalence study

We calculated mean prevalence rates for each country and assigned a number from 1 to 4 to each country based on the quartile of prevalence to which the country belonged.

We calculated European annual prevalence rates and analysed their trend over time, displaying the results in a two-way plot.

We correlated mean prevalence rates of each country to their relative mean mortality rates using Pearson correlation.

## Results

### Mortality study

We extracted data from 24 EU countries from 2013 to 2018.

The overall European population ranged from 468 million (230 million (49%) males and 238 million (51%) females) residents in 2013 to 414 million (203 million (49%) males and 211 million (51%) females) residents in 2018 (supplementary material S6). In 2018, missing data from France accounted for 67 million people, with 32 million (48%) males and 35 million (52%) females. As a result, the overall European population was estimated at 481 million residents. The overall European population in 2017 used for direct standardisation was estimated at 480 million residents (supplementary material S4).

We extracted a total of 104 767 IPF-CS (ICD-10 code J84.1) deaths from 2013 and 2018 across all 24 of the EU countries. 63 784 (61%) were males and 40 983 (39%) were females. The crude overall mortality rate in the 2013–2018 calendar period was 3.73 (95% CI 3.71–3.75) per 100 000 person-years, rising from 3.45 (95% CI 3.39–3.50) in 2013 to 4.11 (95% CI 4.05–4.17) in 2018 (table 1).

**TABLE 1:** Deaths and crude mortality rates (95% CI) per 100 000 person-years for idiopathic pulmonary fibrosis-clinical syndrome in 24 European Union countries in 2013–2018

	2013		2014		2015		2016		2017		2018		Overall (2013–2018)	
	Deaths	Crude rate	Deaths	Crude rate	Deaths	Crude rate	Deaths	Crude rate	Deaths	Crude rate	Deaths	Crude rate	Deaths	Crude rate
**Austria**	149	1.76 (1.50–2.70)	169	1.99 (1.70–2.31)	170	2.05 (1.75–2.38)	158	1.88 (1.60–2.20)	171	2.94 (2.52–3.42)	197	2.23 (1.93–2.57)	1014	2.10 (1.97–2.23)
**Belgium**	314	2.82 (2.52–3.15)	271	2.42 (2.14–2.73)	298	2.65 (2.36–2.97)	347	3.07 (2.75–3.41)	319	2.90 (2.59–3.24)	337	4.52 (4.05–5.03)	1886	2.98 (2.85–3.12)
**Bulgaria**	31	0.71 (0.50–1.01)	23	0.57 (0.36–0.86)	12	0.24 (0.10–0.36)	10	0.29 (0.14–0.54)	12	0.20 (0.10–0.35)	18	0.44 (0.26–0.70)	106	0.38 (0.31–0.46)
**Croatia**	18	0.46 (0.19–0.73)	18	2.82 (1.67–4.46)	20	1.86 (1.14–2.87)	22	0.91 (0.57–1.39)	19	0.77 (0.47–1.21)	25	1.75 (1.14–2.59)	122	1.01 (0.85–1.22)
**Denmark**	75	1.34 (1.07–1.68)	88	1.56 (1.25–1.93)	85	1.55 (1.24–1.92)	85	1.49 (1.19–1.84)	96	2.70 (2.19–3.29)	123	2.20 (1.83–2.62)	552	1.75 (1.61–1.90)
**Estonia**	3	0.65 (0.21–2.01)	9	1.55 (0.71–2.94)	5	7.26 (2.36–16.95)	6	1.22 (0.45–2.65)	6	1.16 (0.42–2.52)	17	1.51 (0.88–2.42)	46	1.42 (1.04–1.89)
**Finland**	246	6.75 (5.96–7.65)	240	6.68 (5.86–7.58)	256	6.93 (6.11–7.84)	310	8.35 (7.44–9.33)	252	7.09 (6.25–8.03)	289	7.70 (6.84–8.64)	1593	7.26 (6.91–7.62)
**France**	1616	2.46 (2.35–2.59)	1723	2.60 (2.48–2.73)	1854	2.79 (2.66–2.92)	1871	2.81 (2.68–2.94)	1878	2.81 (2.69–2.94)	NA	NA	8942	2.70 (2.64–2.75)
**Germany**	2823	3.51 (3.38–3.64)	2806	3.47 (3.35–3.61)	2940	3.62 (3.49–3.75)	3075	3.74 (3.61–3.88)	3151	3.82 (3.69–3.95)	3146	3.80 (3.67–3.94)	17 941	3.66 (3.61–3.72)
**Greece**	N/A	0.33 (0.05–2.32)	376	3.44 (3.10–3.81)	419	3.86 (3.51–4.25)	394	3.79 (3.42–4.18)	415	3.85 (3.49–4.24)	386	3.59 (3.24–3.97)	1991	3.69 (3.53–3.85)
**Hungary**	164	1.65 (1.42–1.93)	150	1.52 (1.29–1.78)	176	1.79 (1.53–2.07)	178	1.81 (1.55–2.10)	186	1.90 (1.64–2.19)	214	2.25 (1.96–2.58)	1068	1.82 (1.71–1.93)
**Ireland**	276	5.99 (5.32–6.74)	311	6.71 (5.98–7.49)	309	10.72 (9.50–11.98)	317	6.71 (5.99–7.49)	352	12.42 (11.16–13.79)	360	11.96 (10.75–13.26)	1925	8.48 (8.10–8.87)
**Italy**	2000	3.35 (3.21–3.50)	2022	3.33 (3.18–3.47)	2148	3.53 (3.39–3.69)	2105	3.47 (3.32–3.62)	2365	3.90 (3.75–4.06)	2480	4.10 (3.94–4.26)	13 120	3.61 (3.55–3.68)
**Luxembourg**	21	4.50 (2.94–6.91)	24	5.11 (3.28–7.61)	17	3.56 (2.07–5.69)	15	5.94 (3.33–9.80)	19	3.91 (2.35–6.10)	22	4.21 (2.64–6.37)	118	4.41 (3.65–5.28)
**Latvia**	8	0.79 (0.39–1.58)	5	4.07 (1.32–9.49)	10	2.90 (1.39–5.33)	5	0.69 (0.22–1.60)	7	2.52 (1.01–5.19)	11	0.72 (0.36–1.28)	46	1.14 (0.84–1.53)
**Netherlands**	445	2.64 (2.40–2.89)	438	2.60 (2.36–2.86)	513	3.04 (2.78–3.31)	494	3.01 (2.75–3.29)	488	2.86 (2.61–3.12)	541	3.15 (2.89–3.43)	2917	2.88 (2.78–2.99)
**Poland**	320	0.84 (0.75–0.94)	349	0.92 (0.82–1.02)	342	0.90 (0.80–1.00)	363	0.96 (0.86–1.06)	354	0.93 (0.84–1.03)	312	0.82 (0.73–0.92)	2040	0.89 (0.86–0.93)
**Portugal**	304	2.90 (2.58–3.24)	325	3.12 (2.79–3.47)	353	3.40 (3.06–3.78)	395	3.82 (3.45–4.22)	345	3.35 (3.00–3.72)	338	3.28 (2.94–3.65)	2060	3.31 (3.17–3.46)
**Romania**	96	0.48 (0.39–0.59)	84	0.42 (0.34–0.52)	105	0.54 (0.44–0.66)	131	0.66 (0.55–0.79)	111	0.56 (0.46–0.68)	124	0.63 (0.53–1.58)	651	0.55 (0.51–0.60)
**Slovenia**	45	3.58 (2.61–4.78)	48	2.51 (1.85–3.33)	45	3.56 (2.59–4.76)	58	2.99 (2.27–3.86)	54	4.78 (3.59–6.24)	76	3.96 (3.12–4.96)	326	3.46 (3.09–3.86)
**Slovakia**	35	0.67 (0.48–0.93)	44	0.81 (0.59–1.09)	41	0.76 (0.55–1.03)	50	0.96 (0.71–1.26)	41	0.81 (0.58–1.10)	48	1.39 (1.03–1.85)	259	0.87 (0.77–0.98)
**Spain**	1883	4.03 (3.85–4.22)	1903	4.09 (3.91–4.28)	2035	4.38 (4.19–4.58)	2045	4.40 (4.21–4.60)	2102	4.52 (4.33–4.72)	2022	4.33 (4.15–4.53)	11 990	4.29 (4.22–4.37)
**Sweden**	391	6.16 (5.57–6.81)	416	4.31 (3.91–4.75)	105	4.83 (4.41–5.29)	131	4.85 (4.42–5.31)	486	4.86 (4.44–5.31)	497	4.91 (4.49–5.36)	2725	4.92 (4.73–5.11)
**UK**	4886	7.65 (7.43–7.86)	5035	7.82 (7.61–8.04)	5261	8.11 (7.90–8.33)	5433	8.31 (8.09–8.53)	5271	8.01 (7.79–8.22)	5443	8.21 (8.00–8.43)	31 329	8.02 (7.93–8.11)
**All Europe**	16 148	3.45 (3.39–3.50)	16 877	3.51 (3.46–3.56)	17 885	3.72 (3.66–3.77)	18 331	3.79 (3.73–3.84)	18 500	3.85 (3.79–3.91)	17 026	4.11 (4.05–4.17)	104 767	3.73 (3.71–3.75)

NA: not available.

We found geographical differences across the European countries, with the highest overall crude rates registered in Ireland (8.48, 95% CI 8.10–8.87 per 100 000 person-years), the UK (8.02, 95% CI 7.93–8.11) and Finland (7.26, 95% CI 6.91–7.62), followed by Sweden (4.92, 95% CI 4.73–5.11), Luxembourg (4.41, 95% CI 3.65–5.28) and Spain (4.29, 95% CI 4.22–4.37). The lowest overall crude rates were detected in Bulgaria (0.38, 95% CI 0.31–0.46 per 100 000 person-years), Slovakia (0.87, 95% CI 0.77–0.98) and Romania (0.89, 95% CI 0.86–0.93). We found middle rates in Portugal (3.46, 95% CI 3.09–3.86 per 100 000 person-years), Italy (3.61, 95% CI 3.55–3.68), Germany (3.66, 95% CI 3.61–3.72) and Greece (3.69, 95% CI 3.53–3.85) (table 1).

**FIGURE 1:**
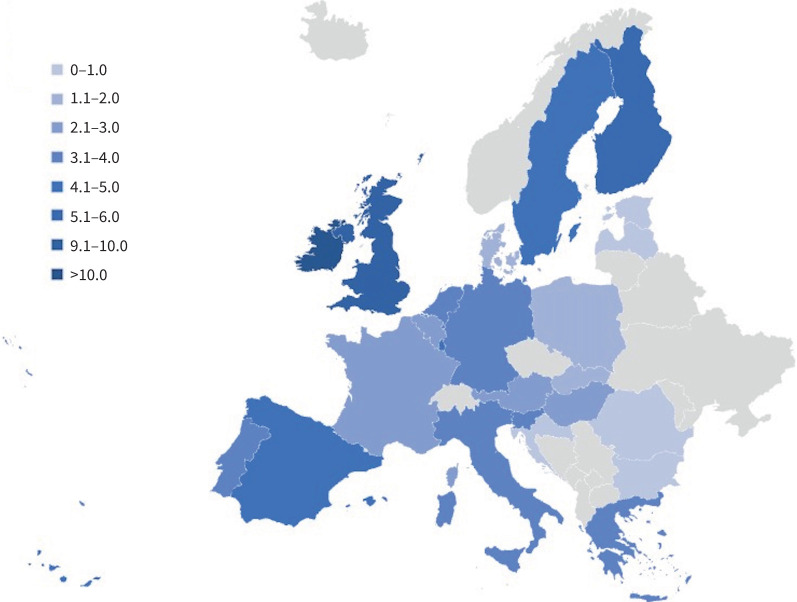
Geographical distribution of idiopathic pulmonary fibrosis-clinical syndrome average age- and sex-standardised mortality rates in 24 European Union countries in 2015–2017. Mortality rates reported as number of deaths per 100 000 person-years.

**FIGURE 2:**
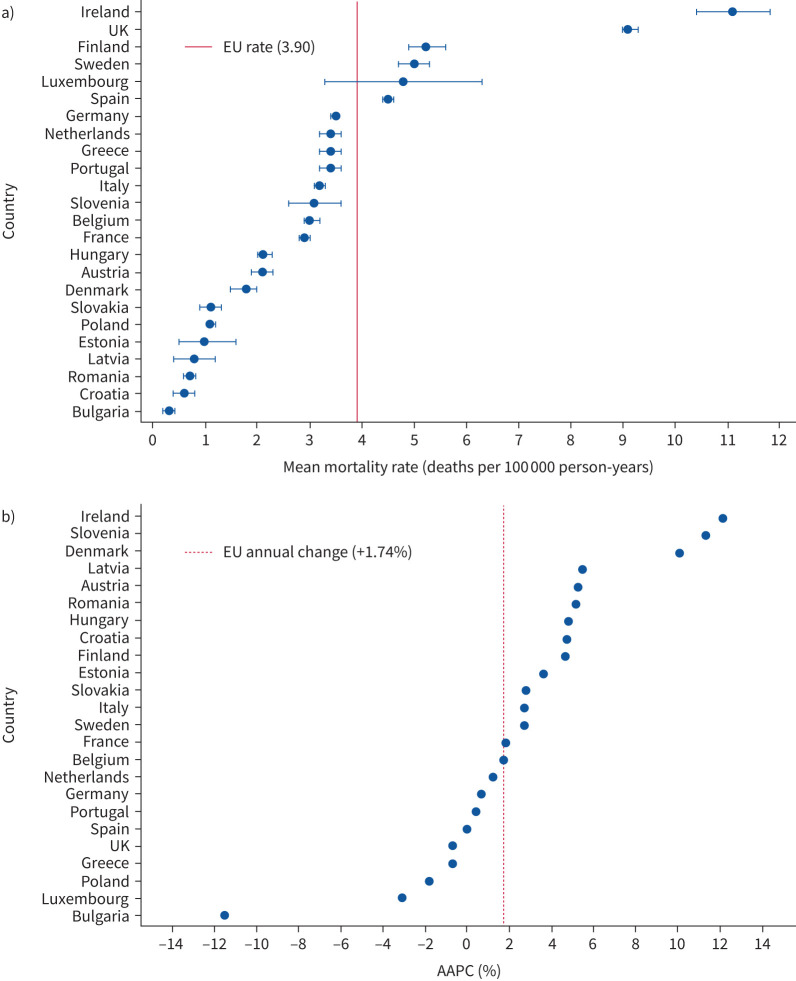
a) Average age- and sex-standardised mortality rates (95% CI) of idiopathic pulmonary fibrosis-clinical syndrome (IPF-CS) in 24 European Union (EU) countries in 2015–2017. b) Average percent annual change (AAPC) in IPF-CS mortality rate in 24 EU countries in 2013–2018.

The overall standardised mortality rate in EU countries over this period was 3.90, 95% CI 3.80–3.90) per 100 000 person-years, with the rate raising from 3.70 (95% CI 3.70–3.80) in 2013 to 4.00 (95% CI 3.90–4.10) in 2018. Standardised rates for each European country followed similar patterns to the crude rates and the inter-country differences were not changed by the standardisation process (table 2 and figures 1 and 2a).

**TABLE 2:** Age- and sex-standardised mortality rates for idiopathic pulmonary fibrosis-clinical syndrome in 24 European Union countries in 2013–2018

	Standardised mortality rate per 100 000 person-years (95% CI)							AAPC (%) (95% CI)
	2013	2014	2015	2016	2017	2018	Overall (2013–2018)	
**Austria**	2.00 (1.70–2.30)	2.20 (1.90–2.60)	2.20 (1.90–2.50)	2.00 (1.70–2.30)	2.10 (1.70–2.40)	2.30 (2.00–2.70)	2.10 (2.00–2.30)	5.25 (−3.84–15.34)
**Belgium**	3.10 (2.80–3.50)	2.70 (2.30–3.00)	2.90 (2.50–3.20)	3.30 (2.90–3.60)	3.00 (2.60–3.30)	3.10 (2.80–3.40)	3.00 (2.90–3.10)	1.75 (−5.32–9.76)
**Bulgaria**	0.50 (0.30–0.60)	0.30 (0.20–0.50)	0.20 (0.10–0.30)	0.10 (0.10–0.20)	0.20 (0.10–0.30)	0.30 (0.10–0.40)	0.40 (0.30–0.50)	−11.53 (−32.95–10.03)
**Croatia**	0.40 (0.20–0.70)	0.50 (0.30–0.80)	0.50 (0.30–0.70)	0.60 (0.30–0.80)	0.50 (0.20–0.70)	0.60 (0.40–0.90)	0.70 (0.50–0.80)	4.72 (−10.47–23.87)
**Denmark**	1.60 (1.20–1.90)	1.80 (1.40–2.20)	1.70 (1.30–2.00)	1.70 (1.30–2.00)	1.90 (1.50–2.20)	2.30 (1.90–2.80)	1.90 (1.70–2.00)	10.07* (0.84–20.59)
**Estonia**	0.20 (0.00–0.50)	0.70 (0.30–1.20)	0.50 (0.10–0.90)	0.50 (0.10–0.80)	0.50 (0.10–0.90)	1.50 (0.80–2.30)	1.70 (1.10–2.20)	3.60 (−18.40.70)
**Finland**	5.10 (4.40–5.70)	4.80 (4.20–5.40)	5.10 (4.40–5.70)	6.00 (5.30–6.60)	4.70 (4.10–5.30)	5.30 (4.60–5.90)	5.20 (4.90–5.40)	4.68 (−6.78–18.05)
**France**	2.60 (2.50–2.80)	2.80 (2.60–2.90)	2.90 (2.80–3.00)	2.90 (2.80–3.00)	2.80 (2.70–2.90)	NA	2.80 (2.80–2.90)	1.84 (−2.31–6.22)
**Germany**	3.40 (3.30–3.60)	3.40 (3.20–3.50)	3.40 (3.30–3.50)	3.50 (3.40–3.60)	3.50 (3.40–3.60)	3.40 (3.30–3.50)	3.40 (3.40–3.50)	0.72 (−0.56–2.09)
**Greece**	NA	3.30 (2.90–3.60)	3.60 (3.20–3.90)	3.30 (3.00–3.60)	3.40 (3.10–3.70)	3.20 (2.80–3.50)	3.30 (3.20–3.50)	−0.72 (−9.63–9.17)
**Hungary**	2.00 (1.70–2.30)	1.80 (1.50–2.10)	2.10 (1.80–2.50)	2.10 (1.80–2.40)	2.20 (1.90–2.50)	2.50 (2.10–2.80)	2.10 (2.00–2.30)	4.81 (−0.77–11.18)
**Ireland**	10.30 (9.10–11.60)	11.30 (10.10–12.60)	10.90 (9.70–12.10)	10.70 (9.50–11.80)	11.70 (10.50–13.00)	11.40 (10.20–12.60)	11.10 (10.60–11.60)	12.16 (1.41–28.63)
**Italy**	3.10 (3.00–3.20)	3.10 (2.90–3.20)	3.20 (3.00–3.30)	3.10 (2.90–3.20)	3.40 (3.20–3.50)	3.50 (3.30–3.60)	3.20 (3.20–3.30)	2.74* (0.73–4.90)
**Luxembourg**	4.60 (2.60–6.60)	5.20 (3.10–7.30)	3.60 (1.90–5.40)	3.50 (1.70–5.30)	3.90 (2.10–5.60)	4.50 (2.60–6.40)	5.70 (4.50–6.90)	−3.10 (−13.34–7.69)
**Latvia**	0.40 (0.10–0.60)	0.30 (0.00–0.70)	0.60 (0.20–1.00)	0.30 (0.00–0.50)	0.40 (0.10–0.70)	0.60 (0.30–1.00)	1.00 (0.60–1.30)	5.45 (−42.23–95.65)
**Netherlands**	3.30 (3.00–3.60)	3.10 (2.80–3.40)	3.60 (3.30–3.90)	3.30 (3.00–3.60)	3.20 (2.90–3.50)	3.50 (3.20–3.80)	3.30 (3.20–3.50)	1.17 (−2.68–5.28)
**Poland**	1.10 (1.00–1.20)	1.20 (1.00–1.30)	1.10 (1.00–1.20)	1.20 (1.10–1.30)	1.10 (1.00–1.20)	1.00 (0.90–1.10)	1.10 (1.10–1.20)	−1.80 (−6.75–3.33)
**Portugal**	3.00 (2.70–3.40)	3.20 (2.80–3.50)	3.40 (3.00–3.70)	3.70 (3.40–4.10)	3.20 (2.80–3.50)	3.10 (2.70–3.40)	3.30 (3.10–3.40)	0.44 (5.59–6.91)
**Romania**	0.60 (0.40–0.70)	0.50 (0.40–0.60)	0.60 (0.50–0.70)	0.80 (0.60–0.90)	0.60 (0.50–0.80)	0.70 (0.60–0.80)	0.60 (0.60–0.70)	5.17 (−6.09–19.42)
**Slovenia**	2.60 (1.90–3.40)	2.90 (2.10–3.80)	2.50 (1.80–3.20)	3.30 (2.40–4.10)	2.90 (2.10–3.70)	4.00 (3.10–4.90)	3.20 (2.90–3.60)	11.35 (−0.29–25.53)
**Slovakia**	0.90 (0.60–1.30)	1.20 (0.80–1.60)	1.00 (0.70–1.30)	1.20 (0.90–1.60)	1.00 (0.70–1.30)	1.10 (0.80–1.40)	1.10 (1.00–1.30)	2.85 (−6.27–12.98)
**Spain**	4.40 (4.20–4.60)	4.30 (4.10–4.50)	4.50 (4.30–4.70)	4.40 (4.20–4.60)	4.50 (4.30–4.70)	4.20 (4.10–4.40)	4.40 (4.30–4.50)	−0.04 (−2.63–2.71)
**Sweden**	4.40 (3.90–4.80)	4.50 (4.10–5.00)	5.10 (4.60–5.50)	4.90 (4.40–5.30)	5.00 (4.60–5.50)	5.00 (4.60–5.50)	4.80 (4.70–5.00)	2.69* (0.52–4.94)
**UK**	8.90 (8.70–9.20)	9.00 (8.70–9.20)	9.20 (9.00–9.50)	9.30 (9.10–9.60)	8.90 (8.60–9.10)	8.90 (8.70–9.20)	9.00 (8.90–9.10)	−0.72 (−9.62–6.17)
**All Europe**	3.70 (3.70–3.80)	3.70 (3.70–3.80)	3.90 (3.80–3.90)	3.90 (3.90–4.00)	3.80 (3.80–3.90)	4.00 (3.90–4.10)	3.90 (3.80–3.90)	1.74* (0.91–2.59)

AAPC: average annual percent change; NA: not available. *: statistically significant (p<0.05).

**FIGURE 3:**
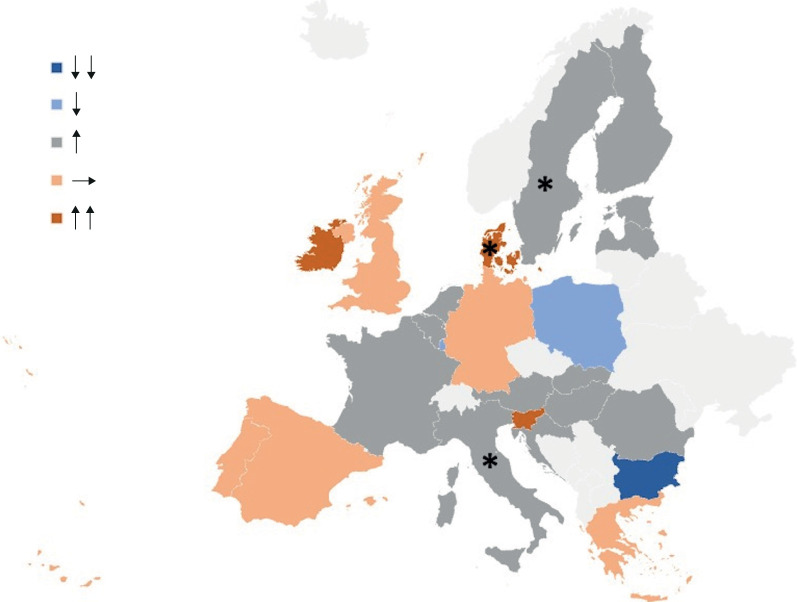
Geographical distribution of idiopathic pulmonary fibrosis-clinical syndrome mortality rate trends in 24 European Union countries from 2013 to 2018. Upward-pointing double arrows (↑↑) and a single upward arrow (↑) indicate overall increasing trends. Downward-pointing double arrows (↓↓) and a single downward arrow (↓) signify overall decreasing trends. A horizontal arrow (→) denotes stability in trends. *: statistically significant variations (p<0.05).

Mean standardised mortality rates for the entire study period (2013–2018) are reported in supplementary material S7.

Overall mortality rates were lower in females compared to males (adjusted mortality rate ratio 0.41, 95% CI 0.41–0.42; p<0.01). There was a substantial increased mortality risk with ageing, with a mean increase of 100% between one age category and the next (adjusted mortality rate ratio 2.03, 95% CI 2.02–2.03; p<0.01).

After controlling for the effects of sex and age, we observed an increase in mortality rates year on year in the study period, with an AAPC of 1.74% (95% CI 0.91–2.59%). The highest annual increase was found in the elderly (AAPC 2.23% (95% CI 0.84–3.72%) in those aged >85 years) (table 3).

**TABLE 3:** Overall age- and sex-standardised mortality rates for idiopathic pulmonary fibrosis-clinical syndrome in Europe in 2013–2018: age and sex strata data

	Standardised mortality rate per 100 000 person-years (95% CI)							AAPC (%) (95% CI)
	2013	2014	2015	2016	2017	2018	Overall (2013–2018)	
**Total population**	3.70 (3.70–3.80)	3.70 (3.70–3.80)	3.90 (3.80–3.90)	3.90 (3.90–4.00)	3.80 (3.80–3.90)	4.00 (3.90–4.10)	3.90 (3.80–3.90)	1.74* (0.91–2.59)
**Sex**								
Female	2.90 (2.90–3.00)	2.90 (2.80–2.90)	3.00 (2.90–3.10)	3.10 (3.00–3.10)	3.00 (2.90–3.00)	3.00 (3.00–3.10)	2.90 (2.90–3.00)	1.20 (−0.03–2.46)
Male	4.20 (4.10–4.30)	4.30 (4.30–4.40)	4.60 (4.50–4.70)	4.60 (4.60–4.70)	4.80 (4.70–4.80)	5.20 (5.10–5.30)	4.70 (4.70–4.80)	1.61* (0.53–2.74)
**Age group**								
0–54 years	0.10 (0.10–0.10)	0.10 (0.10–0.10)	0.10 (0.10–0.10)	0.10 (0.10–0.10)	0.10 (0.10–0.10)	0.10 (0.10–0.10)	0.10 (0.10–0.10)	−0.40 (−9.50–9.51)
55–59 years	1.10 (1.00–1.30)	1.10 (1.00–1.20)	1.20 (1.10–1.30)	1.10 (1.00–1.30)	1.10 (1.00–1.20)	1.00 (0.90–1.10)	1.10 (1.10–1.20)	−1.58 (−6.29–3.23)
60–64 years	2.60 (2.50–2.80)	2.60 (2.40–2.80)	2.50 (2.40–2.70)	2.70 (2.50–2.90)	2.50 (2.40–2.70)	2.60 (2.40–2.80)	2.60 (2.50–2.70)	−0.05 (−1.32–1.18)
65–69 years	5.30 (5.00–5.60)	5.50 (5.30–5.80)	5.40 (5.10–5.60)	5.40 (5.10–5.60)	5.00 (4.80–5.30)	5.20 (4.90–5.50)	5.30 (5.20–5.40)	−1.15 (2.49–0.18)
70–74 years	10.60 (10.20–11.10)	11.10 (10.70–11.60)	10.40 (10.00–10.80)	10.70 (10.30–11.20)	10.10 (9.70–10.50)	11.70 (11.20–12.20)	10.80 (10.60–11.00)	0.71 (−3.70–5.24)
75–79 years	18.80 (18.20–19.50)	19.00 (18.40–19.60)	19.80 (19.20–20.50)	19.80 (19.20–20.50)	19.50 (18.90–20.20)	20.00 (19.30–20.70)	19.50 (19.30–19.80)	1.11* (0.01–2.22)
80–84 years	28.20 (27.20–29.10)	27.90 (27.00–28.80)	30.50 (29.60–31.40)	30.60 (29.60–31.40)	30.30 (29.40–31.20)	30.80 (29.90–31.80)	29.70 (29.40–30.10)	2.02* (0.10–3.99)
>85 years	39.30 (38.10–40.50)	38.10 (37.00–39.20)	40.70 (39.60–41.90)	40.60 (39.50–41.80)	42.10 (41.00–43.20)	43.20 (42.00–44.40)	40.70 (40.20–41.20)	2.23* (0.84–3.72)

AAPC: average annual percent change. *: statistically significant (p<0.05).

We found that annual mortality rates were increasing in most European countries, with significant rising trends in Denmark, Italy and Sweden, whereas we saw the greatest relative decrease in mortality in Bulgaria (table 2 and figure 2b).

We observed distinct patterns of association between mortality rates and trends over time in the European countries. Some countries exhibited both elevated mortality rates and an increasing trend, such as Ireland and Sweden. Conversely, Bulgaria and Poland demonstrated low mortality rates coupled with a decreasing trend. The UK exhibited high mortality rates, but no increase over time. Denmark, Hungary, Romania, Slovenia and Slovakia displayed low mortality rates, yet experienced a rising trend over time. Finally, Luxembourg showed a high mortality rate initially which then fell in the middle years before climbing again (figure 2).

In our broader analysis using the J84 codes, the patterns of morality rate were very similar, but slightly higher. For example, the overall standardised mortality rate during our study period was 4.60 per 100 000 person-years compared to 3.90 using the narrow definition (supplementary material S8–S11).

Crude and standardised IPF-CS mortality rates both of broad and narrow codeset bands for 2019 and 2020 are provided in supplementary material S12.

### Prevalence study

The overall European ILD and sarcoidosis prevalence rate increased from 91.35 (95% CI 79.83–103.69) cases per 100 000 in 2013 to 97.67 (95% CI 83.66–112.79) in 2018 (supplementary material S13 and S14).

We detected the highest prevalence rates in Finland, Ireland, Italy, Spain, Sweden and the UK, whereas Bulgaria, Estonia, France, Greece and Slovenia had the lowest prevalence rates (supplementary material S15 and S16).

We found an overall increasing prevalence trend across Europe (annual prevalence rate change 0.08 (95% CI 0.01–0.16) cases per 100 000) (supplementary material S17).

Most European countries exhibited a rising prevalence trend, with the highest increases in Bulgaria, Greece, Ireland, the Netherlands, Poland and Slovakia. Austria and Latvia showed decreasing prevalence trends (supplementary material S9).

### Correlation between prevalence and mortality

We found that the overall correlation between the prevalence rates and the mortality rates of each European country was moderate (r=0.54).

Finland, Ireland, Sweden, Spain and the UK had both high IPF mortality rates and high ILD and sarcoidosis prevalence rates.

Bulgaria and Slovakia had low IPF mortality rates and low ILD and sarcoidosis prevalence rates.

Luxembourg exhibited high IPF mortality rates with low ILD and sarcoidosis prevalence rates. Conversely, Italy showed middle IPF mortality rates with high prevalence rates.

Most countries with middle mortality rates also had middle prevalence rates.

## Discussion

In our mortality study, we found the overall age- and sex-standardised mortality rate for IPF-CS in Europe between 2013 and 2018 was 3.90 per 100 000 person-years (4.60 per 100 000 person-years for males and 2.90 per 100 000 person-years for females). We found evidence that this rate increased over time from 2013 to 2018 (AAPC 1.74%, 95% CI 0.91–2.59%) across 24 EU countries. This equates to more than 17 000 recorded deaths each year in Europe between 2013 and 2018.

We found marked differences between the countries, with the highest rates in Ireland, the UK, Luxembourg and Finland, followed by Sweden and Spain, and lower rates in Bulgaria, Romania and Croatia. We detected increases over time in IPF-CS mortality rates in Denmark, Italy and Sweden, whereas Bulgaria showed a decrease over time. Registered deaths were highest in men and the elderly over all the analysed years and in all countries [1, 8, 9].

The results of our second, broader analysis using a wider range of IPF codes were consistent with those of the main analysis, but with ∼15% more deaths each year.

In our prevalence study, we found that the ILD prevalence rate is increasing in Europe (annual prevalence rate change 0.08, 95% CI 0.01–0.16 cases per 100 000) and in most European countries, but again with substantial inter-country differences. Countries exhibiting the highest prevalence rates generally also had the highest mortality rates and, conversely, countries with the lowest mortality rates generally had the lowest prevalence rates, although in statistical terms this correlation was only moderate.

The main strengths of our study include the intrinsic value of the EUROSTAT dataset, the large number of deaths in our dataset and the wide range of European countries included. We deliberately chose the time period for our study to ensure we removed problems in coding due to the coronavirus disease 2019 pandemic.

The main potential weaknesses of our study are the reliability of a clinical diagnosis of IPF and the recording of these diagnoses in death registries. We believe that an over-recording of IPF deaths is less likely than under-recording because it is unlikely that a diagnosis of IPF will be recorded on a death certificate unless this diagnosis has been made in life by a secondary care specialist or by a multidisciplinary team. Previously, Johnston *et al*. [10] showed that for a cohort of people with known lung fibrosis in the UK, less than half had this diagnosis recorded on their death certificate, and for only 15% was this recorded as the underlying cause of death.

Our GBD data also have limitations, partly because the data capture all ILD including sarcoidosis. However, the correlation between our prevalence and mortality datasets, although only moderate, provides reassurance that death certificate data is capturing diagnosed disease in most countries. However, the question of how much disease is undiagnosed remains, as well as how this varies by country.

The increasing IPC-CS mortality rate across Europe has been previously reported by Marshall *et al*. [2], who assessed the IPF mortality rate across 17 EU countries in 2001–2013, using WHO data. They reported a median (interquartile range (IQR)) mortality of 3.8 (1.37–5.30) and 1.5 (0.64–2.02) per 100 000 for males and females, respectively. Thus, their results for overall mortality rates are similar to ours. Their study was limited, however, as it did not provide an overall analysis of IPF mortality rate over time and only analysed data from 17 countries.

Salciccioli *et al*. [11] performed a more recent study, analysing mortality trends in ILDs up to 2017, using the GBD dataset. They observed an increasing incidence and a decreasing mortality trend in almost all the examined countries, which is in contrast to our results. However, as all ILDs and sarcoidosis were grouped together in their analysis, their mortality data are not directly comparable to ours.

In a UK study of death certificates, Navaratnam *et al*. [1] found an age-standardised mortality rate of 8.26 per 100 000 person-years in 2016 with a 5% mean annual increase from 1979 to 2016. Our UK rates are similar to these, but we observed an apparent plateauing in the increasing trend of the IPF-CS mortality rate in the UK (AAPC −0.72%, 95% CI −9.62–6.17%). This plateauing, and the high mortality rate in the UK, suggest that the recorded mortality rate in the UK may now be approaching the true mortality rate.

To the best of our knowledge no pan-European studies quantifying the incidence and prevalence of IPF have yet been performed. Nevertheless, our GBD prevalence study showed an increasing ILD prevalence rate over time in Europe and in most European countries. Although not specific for IPF, this might suggest that part of the IPF mortality increase may be related to a rising disease incidence and prevalence.

The recent introduction of nintedanib and pirfenidone to treat lung fibrosis should reduce mortality from this disease in time [12–14]; however, our data are probably too close to the introduction of these treatments to show any beneficial impacts.

There are a number of possible explanations for the geographical differences in mortality we found. First, there may be true differences in mortality between countries in Europe. Second, there may be social and healthcare differences that lead to different rates of diagnosis and subsequent case ascertainment. Third, there may be differences in the practice of death certificate recording.

In terms of social and demographic factors, the differences in smoking habits, population age structures and ethnicities across Europe do not appear to be marked enough to explain the differences in mortality we have seen [15–17].

We did find some moderate correlation between country IPF mortality rate and the prevalence of ILDs, which suggests that some of the variation in mortality we observed may reflect differences in disease prevalence, or at least differences in the amount of diagnosed disease.

Moor *et al*. [18] did not find significant divergences in terms of healthcare for people with ILD or in antifibrotic therapy availability across 14 European countries. However, they detected a great variability of multidisciplinary team composition that might lead to different ascertainment of cases.

Finally, the practice of death certification itself varies greatly across European countries [19], and so may possibly amplify inter-country differences in IPF mortality rates.

For these reasons, we believe that a combination of under-diagnosis of IPF and under-recording of IPF deaths is the likely reason for the geographical differences we have observed. If these assumptions are true, and the true incidence in Europe is close to the incidence rate for Ireland and the UK, then the actual annual number of deaths from IPF in Europe is more likely to be close to 40 000 rather than the 17 000 recorded.

### Conclusions

IPF-CS is an important cause of mortality across Europe and a public health concern, with more than 17 000 deaths recorded each year, and the burden is increasing. We believe that the under-diagnosis of IPF is the most important driver of this variation. If all countries in Europe had a similar mortality rate to the UK and Ireland, we estimate that there would actually be more than 40 000 deaths from IPF each year in Europe.

## Supplementary material

10.1183/13993003.02080-2023.Supp1 **Please note:** supplementary material is not edited by the Editorial Office, and is uploaded as it has been supplied by the author.
Supplementary material ERJ-02080-2023.Supplement

## Shareable PDF

10.1183/13993003.02080-2023.Shareable1 This one-page PDF can be shared freely online.
Shareable PDF ERJ-02080-2023.Shareable

## Data Availability

The data that support the findings of this study are available from the EUROSTAT and GBD websites. These data were derived from the following publicly available resources: https://ec.europa.eu/eurostat and www.healthdata.org/research-analysis/gbd

## References

[1] Navaratnam V, Hubbard RB. The mortality burden of idiopathic pulmonary fibrosis in the United Kingdom. Am J Respir Crit Care Med 2019; 200: 256–258. doi:10.1164/rccm.201902-0467LE 30973756

[2] Marshall DC, Salciccioli JD, Shea BS, et al. Trends in mortality from idiopathic pulmonary fibrosis in the European Union: an observational study of the WHO mortality database from 2001–2013. Eur Respir J 2018; 51: 1701603. doi:10.1183/13993003.01603-2017 29348182

[3] European Institute of Statistics. Welcome to Eurostat: the home of high quality statistics and data in Europe. 2023. https://ec.europa.eu/eurostat Date last accessed: 12 September 2023.

[4] Morgan A, Gupta RS, George PM, et al. Validation of the recording of idiopathic pulmonary fibrosis in routinely collected electronic healthcare records in England. BMC Pulm Med 2023; 23: 256. doi:10.1186/s12890-023-02550-0 37434192 PMC10337174

[5] Navaratnam V, Fogarty AW, Glendening R, et al. The increasing secondary care burden of idiopathic pulmonary fibrosis: hospital admission trends in England from 1998 to 2010. Chest 2013; 143: 1078–1084. doi:10.1378/chest.12-0803 23188187

[6] Navaratnam V, Fleming KM, West J, et al. The rising incidence of idiopathic pulmonary fibrosis in the UK. Thorax 2011; 66: 462–467. doi:10.1136/thx.2010.148031 21525528

[7] Benchimol EI, Smeeth L, Guttmann A, et al. The REporting of studies Conducted using Observational Routinely-collected health Data (RECORD) statement. PLoS Med 2015; 12: e1001885. doi:10.1371/journal.pmed.1001885 26440803 PMC4595218

[8] Strongman H, Kausar I, Maher TM. Incidence, prevalence, and survival of patients with idiopathic pulmonary fibrosis in the UK. Adv Ther 2018; 35: 724–736. doi:10.1007/s12325-018-0693-1 29644539 PMC5960490

[9] Hutchinson J, Fogarty A, Hubbard R, et al. Global incidence and mortality of idiopathic pulmonary fibrosis: a systematic review. Eur Respir J 2015; 46: 795–806. doi:10.1183/09031936.00185114 25976683

[10] Johnston I, Britton J, Kinnear W, et al. Rising mortality from cryptogenic fibrosing alveolitis. Br Med J 1990; 301: 1017–1021. doi:10.1136/bmj.301.6759.1017 2249048 PMC1664054

[11] Salciccioli JD, Marshall DC, Goodall R, et al. Interstitial lung disease incidence and mortality in the UK and the European Union: an observational study, 2001–2017. ERJ Open Res 2022; 8: 00058-2022. doi:10.1183/23120541.00058-2022 35821757 PMC9271755

[12] King TE, Bradford WZ, Castro-Bernardini S, et al. A phase 3 trial of pirfenidone in patients with idiopathic pulmonary fibrosis. N Engl J Med 2014; 370: 2083–2092. doi:10.1056/nejmoa1402582 24836312

[13] Richeldi L, du Bois RM, Raghu G, et al. Efficacy and safety of nintedanib in idiopathic pulmonary fibrosis. N Engl J Med 2014; 370: 2071–2082. doi:10.1056/nejmoa1402584 24836310

[14] Petnak T, Lertjitbanjong P, Thongprayoon C, et al. Impact of antifibrotic therapy on mortality and acute exacerbation in idiopathic pulmonary fibrosis: a systematic review and meta-analysis. Chest 2021; 160: 1751–1763. doi:10.1016/j.chest.2021.06.049 34217681

[15] Reitsma MB, Kendrick PJ, Ababneh E, et al. Spatial, temporal, and demographic patterns in prevalence of smoking tobacco use and attributable disease burden in 204 countries and territories, 1990–2019: a systematic analysis from the Global Burden of Disease Study 2019. Lancet 2021; 397: 2337–2360. doi:10.1016/S0140-6736(21)01169-7 34051883 PMC8223261

[16] Olson AL, Swigris JJ, Lezotte DC, et al. Mortality from pulmonary fibrosis increased in the United States from 1992 to 2003. Am J Respir Crit Care Med 2007; 176: 277–284. doi:10.1164/rccm.200701-044OC 17478620

[17] Dove EP, Olson AL, Glassberg MK. Trends in idiopathic pulmonary fibrosis-related mortality in the United States: 2000–2017. Am J Respir Crit Care Med 2019; 200: 929–931. doi:10.1164/rccm.201905-0958LE 31225965

[18] Moor CC, Wijsenbeek MS, Balestro E, et al. Gaps in care of patients living with pulmonary fibrosis: a joint patient and expert statement on the results of a Europe-wide survey. ERJ Open Res 2019; 5: 00124-2019. doi:10.1183/23120541.00124-2019 31649949 PMC6801215

[19] Millares Martin P. Medical certificate of cause of death: looking for an European single standard. J Forensic Leg Med 2020; 75: 102052. doi:10.1016/j.jflm.2020.102052 32891932

