# Supplementary material for: Mortality trends in idiopathic pulmonary fibrosis in Europe between 2013 and 2018
Source: Eur Respir J. 2024 Aug 22;64(2):2302080. doi: 10.1183/13993003.02080-2023 (PMC11339406; doi:10.1183/13993003.02080-2023)
Supplement: Supplementary file 1 [file ERJ-02080-2023.Supplement.pdf]

Supplementary Materials

Supplementary material S1 page 2

Supplementary material S2 page 4

Supplementary material S3 page 5

Supplementary material S4 page 7

Supplementary material S5 page 8

Supplementary material S6 page 9

Supplementary material S7 page 10

Supplementary material S8 page 11

Supplementary material S9 page 12

Supplementary material S10 page 13

Supplementary material S11 page 14

Supplementary material S12 page 15

Supplementary material S13 page 16

Supplementary material S14 page 17

Supplementary material S15 page 18

Supplementary material S16 page 19

Supplementary material S17 page 20

Supplementary Table S1: the table contains an extract of the entire dataset. We extracted Austrian data from 2013 to 2018 as sample of the entire dataset.

sex	year	country	age_cat	J841_deaths	J84_deaths	pop
male	2013	Austria	0-54y	4	4	2987403
male	2013	Austria	55-59y	2	3	263952
male	2013	Austria	60-64y	7	7	227856
male	2013	Austria	65-69y	8	8	194150
male	2013	Austria	70-74y	22	24	197769
male	2013	Austria	75-79y	22	24	111446
male	2013	Austria	80-84y	12	15	84574
male	2013	Austria	>85y	21	21	56472
female	2013	Austria	0-54y	1	1	2926022
female	2013	Austria	55-59y	1	2	274449
female	2013	Austria	60-64y	3	3	244921
female	2013	Austria	65-69y	6	8	218377
female	2013	Austria	70-74y	2	3	234131
female	2013	Austria	75-79y	5	7	149017
female	2013	Austria	80-84y	14	15	134093
female	2013	Austria	>85y	19	20	147228
male	2014	Austria	0-54y	2	3	2994017
male	2014	Austria	55-59y	1	1	274129
male	2014	Austria	60-64y	5	5	226712
male	2014	Austria	65-69y	7	7	197962
male	2014	Austria	70-74y	27	29	202871
male	2014	Austria	75-79y	15	15	115075
male	2014	Austria	80-84y	26	27	84663
male	2014	Austria	>85y	22	26	59910
female	2014	Austria	0-54y	1	1	2928982
female	2014	Austria	55-59y	2	2	283947
female	2014	Austria	60-64y	2	3	243341
female	2014	Austria	65-69y	6	7	222458
female	2014	Austria	70-74y	6	6	240554
female	2014	Austria	75-79y	17	20	151566
female	2014	Austria	80-84y	14	16	132544
female	2014	Austria	>85y	16	16	149055
male	2015	Austria	0-54y	3	4	3009998
male	2015	Austria	55-59y	2	3	286315
male	2015	Austria	60-64y	6	8	228629
male	2015	Austria	65-69y	6	7	200502
male	2015	Austria	70-74y	19	23	194901
male	2015	Austria	75-79y	25	26	132195
male	2015	Austria	80-84y	27	29	84383
male	2015	Austria	>85y	18	18	63474
female	2015	Austria	0-54y	3	3	2936269
female	2015	Austria	60-64y	4	5	245266
female	2015	Austria	65-69y	8	8	224358
female	2015	Austria	70-74y	4	4	231975
female	2015	Austria	75-79y	13	16	170183
female	2015	Austria	80-84y	15	16	130588
female	2015	Austria	>85y	17	18	151369
male	2016	Austria	0-54y	1	1	3052884
male	2016	Austria	55-59y	2	4	298349
male	2016	Austria	60-64y	6	7	233768
male	2016	Austria	65-69y	17	18	211306
male	2016	Austria	70-74y	17	17	176970
male	2016	Austria	75-79y	20	22	149322
male	2016	Austria	80-84y	21	22	83394
male	2016	Austria	>85y	19	20	66560
female	2016	Austria	0-54y	2	3	2955075
female	2016	Austria	60-64y	4	6	250528
female	2016	Austria	65-69y	4	4	235642
female	2016	Austria	70-74y	10	11	211581
female	2016	Austria	75-79y	18	20	190031
female	2016	Austria	80-84y	6	7	128018
female	2016	Austria	>85y	11	13	152143
male	2017	Austria	0-54y	1	3	3060079
male	2017	Austria	55-59y	7	7	309458
male	2017	Austria	60-64y	6	6	243935
male	2017	Austria	65-69y	3	4	213857
male	2017	Austria	70-74y	17	18	169449
male	2017	Austria	75-79y	35	41	162458
male	2017	Austria	80-84y	19	21	83888
male	2017	Austria	>85y	19	22	69317
female	2017	Austria	55-59y	2	5	313528
female	2017	Austria	60-64y	4	4	261045
female	2017	Austria	65-69y	1	2	239057
female	2017	Austria	70-74y	10	10	201473

female	2017	Austria	75-79y	17	19	205723
female	2017	Austria	80-84y	12	13	126673
female	2017	Austria	>85y	18	20	153622
male	2018	Austria	0-54y	2	2	3052552
male	2018	Austria	55-59y	1	2	321042
male	2018	Austria	60-64y	1	1	253644
male	2018	Austria	65-69y	7	8	211851
male	2018	Austria	70-74y	25	27	173824
male	2018	Austria	75-79y	32	34	168992
male	2018	Austria	80-84y	17	20	85112
male	2018	Austria	>85y	38	44	71501
female	2018	Austria	0-54y	2	2	2954555
female	2018	Austria	55-59y	3	3	323570
female	2018	Austria	60-64y	4	4	269912
female	2018	Austria	65-69y	2	2	236340
female	2018	Austria	70-74y	9	12	206227
female	2018	Austria	75-79y	21	22	214392
female	2018	Austria	80-84y	15	15	125401
female	2018	Austria	>85y	18	19	153352

Supplementary Material S2

Global Burden of Disease dataset

The data utilised for this observational analysis of ILD were sourced from the Global Burden of Disease (GBD) database, which aggregates mortality and disability data pertaining to a range of global health issues. This includes information such as deaths, death rates, years of life lost due to premature mortality, prevalence, and incidence rates.

The GBD methodology is clearly explained on the website (https://www.healthdata.org/data-tools-practices/data-collection).

In summary, the GBD employs a comprehensive approach that incorporates systematic reviews, survey data, disease registries, hospital administrative records, claims data, as well as inpatient and outpatient records, alongside case notifications to estimate disease incidence (for the GBD methodology regarding ILDs and sarcoidosis data collection is provided on pages 888-892 in the following document: https://www.thelancet.com/cms/10.1016/S0140-6736(20)30925-9/attachment/7709ecbd-5dbc-4da6-93b2-3fd0bedc16cc/mmc1.pdf.). Disease classifications within the GBD database adhere to the International Classification of Diseases (ICD) coding system. The collation and dissemination of data are managed by the GBD Collaborators, and the datasets are made publicly accessible for further analysis.

We queried the data on the 1st level cause, selecting the same 24 European Countries, and directly extracted the prevalence rates for each year and for each Country. We used prevalence data for ILDs and sarcoidosis (ICD-10 codes "J84 – *other interstitial lung diseases*" + "D86 – *sarcoidosis*") as a proxy.

We extracted the average annual prevalence rate change for each European Country for the interval 2010-2019, as no more restricted intervals were possible.

Supplementary Material S3

Segmented regression model

The model assesses the overall trends in mortality, initially with no Joinpoints, and tests for significant changes in the model with sequential addition of points where there is significant change in the slope of the line. The model also computes an estimated average annual percent change for each trend by fitting a regression line to the natural logarithm of the rates. The log-linear transformation allows us to approximate normal distributions and, by estimating the annual percentage change, we were able to assess change in mortality trend at a constant percentage per year.

On the Joinpoint software input tabs, we used the following options on the basis of Irimata et al. indications (Irimata KE, Bastian BA, Clarke TC, Curtin SC, Badwe R, Rui P. Guidance for Selecting Model Options in the National Cancer Institute Joinpoint Regression Software. Vital Health Stat 1. 2022 Oct;(194):1-22. PMID: 36255743):

● *Heteroscedastic/correlated errors option (Input File tab)* → we used Standard Error (Provided): this option is the loosest as it allows the errors to be both homoscedastic and heteroscedastic, based on the variance structure provided in the data file. Mortality data are suggested to be treated as uncorrelated as no year-on-year correlation is supposed.

● *Log transformation (Input File tab)* → we applied the log transformation option, as it best suited our need to obtain the relative differences and discuss the APCs, rather than looking at absolute rates.

● *Modelling method and constraints (Method and Parameters tab)* → we adopted the Grid model in this case and used the following constraints: minimum number of observations from a joinpoint to either end of the data = 2; minimum number of observations between two joinpoints = 1; number of points to place between adjacent observed values in the grid search = 0.

● *<u>Number of joinpoints (minimum and maximum number of joinpoints) (Method and Parameters tab)</u>*

→ we used 0 as minimum number of joinpoint and increased to 1 the number of maximum joinpoints (default 0 for less than 7 observation points – years in our case).

● *<u>Model selection method (Method and Parameters tab)</u>* → as we had less than 10 time points, we applied the Bayesian Information Criterion (BIC) model.

Supplementary Material S4 Overall population in Europe in 2017 (reference population), sex distribution for each age group

	Female		*Male*	
	(n)	*(%)*	*(n)*	*(%)*
Total population	241821972		238760288	
Age group (years)				
0-54	156397934	65	166765495	70
55-59	16074241	7	16009169	7
60-64	14924509	6	14748361	6
65-69	14579834	6	13277609	6
70-74	11731077	5	9878339	4
75-79	10744389	4	8279014	3
80-84	8499175	4	5587088	2
> 85	8870813	4	4215213	2

n: number of people

Supplementary Material S5

Direct standardisation method

For direct standardisation we used the STATA command *dstdize* using the following syntax:

`dstdize charvar popvar stratavars, by(groupvars) [dstdize_options]` where charvar is the characteristic (i.e., IPF deaths) to be standardised across different subpopulations identified by groupvars (i.e., country and year), popvar defines the weights used in standardization (i.e., number of residents in each country and in each year), stratavars defines the strata across which the weights are to be averaged in dstdize (i.e., sex and age groups).

Therefore, with this command, it is possible to perform a direct standardisation adjusted for covariates of interest. We used the following code:

dstdize *deaths population* ***age sex*** using ("*reference population*"), by (**country year**).

Crude mortality rates and rate ratios calculation

We calculated the crude mortality rates dividing the number of J84.1 deaths by the total resident population for each year and for each country.

To obtain mortality rate ratios in each country, we compared the adjusted mortality rate of a certain category with the adjusted mortality rate of a selected reference (i.e., male vs female).

Supplementary Material S6 Overall row deaths for idiopathic pulmonary fibrosis-clinical syndrome and overall population in Europe in 2013–2018, age- and sex- stratum data

	2013				2014				2015				2016				2017				2018			
	Row deaths		*Population*		*Row deaths*		*Population*		*Row deaths*		*Population*		*Row deaths*		*Population*		*Row deaths*		*Population*		*Row deaths*		*Population*	
	(n)	*(%)*	*(n)*	*(%)*	*(n)*	*(%)*	*(n)*	*(%)*	*(n)*	*(%)*	*(n)*	*(%)*	*(n)*	*(%)*	*(n)*	*(%)*	*(n)*	*(%)*	*(n)*	*(%)*	*(n)*	*(%)*	*(n)*	*(%)*
Total population	16148		468472680		16877		480956851		17885		480896141		18331		483854733		18500		480582260		17026		414222048	
Sex																								
Male	9664	60	230178364	49	10215	61	235055321	49	10825	61	236565910	49	11111	61	238982812	49	11361	61	238760288	50	10608	62	203144304	49
Female	6484	40	238294316	51	6662	39	245901530	51	7060	39	244330231	51	7220	39	244871921	51	7139	39	241821972	50	6418	38	211077744	51
Age group (years)																								
0-54	386	2	322096859	69	332	2	329593231	69	339	2	327940392	68	382	2	329891609	68	320	2	323163429	67	319	2	274386270	66
55-59	352	2	30935784	7	340	2	31779581	7	387	2	31557737	7	359	2	30855259	6	345	2	32083410	7	294	2	28698313	7
60-64	759	5	29295806	6	752	4	29184857	6	739	4	29457368	6	791	4	29466348	6	753	4	29672870	6	687	4	26818567	6
65-69	1268	8	23750775	5	1411	8	25514025	5	1401	8	25859972	5	1472	8	27555171	6	1406	8	27857443	6	1253	7	24041723	6
70-74	2214	14	20811085	4	2336	14	21021855	4	2223	12	21413892	4	2257	12	20869382	4	2186	12	21609416	4	2310	14	19594322	5
75-79	3270	20	17509183	4	3494	21	18506593	4	3710	21	18766139	4	3767	21	18978162	4	3718	20	19023403	4	3374	20	16865617	4
80-84	3595	22	12922633	3	3724	22	13411361	3	4119	23	13602595	3	4162	23	13565775	3	4267	23	14086263	3	3889	23	12536690	3
> 85	4304	27	11150555	2	4488	27	11945348	2	4967	28	12298046	3	5141	28	12673027	3	5505	30	13086026	3	4900	29	11280546	3

n: number of deaths

Supplementary Material S7: Geographical distribution of idiopathic clinical-syndrome average age- and sex- standardised mortality rates in 24 European Countries in 2013-2018. Figure reports rates as number of deaths per 100,000 person/years.

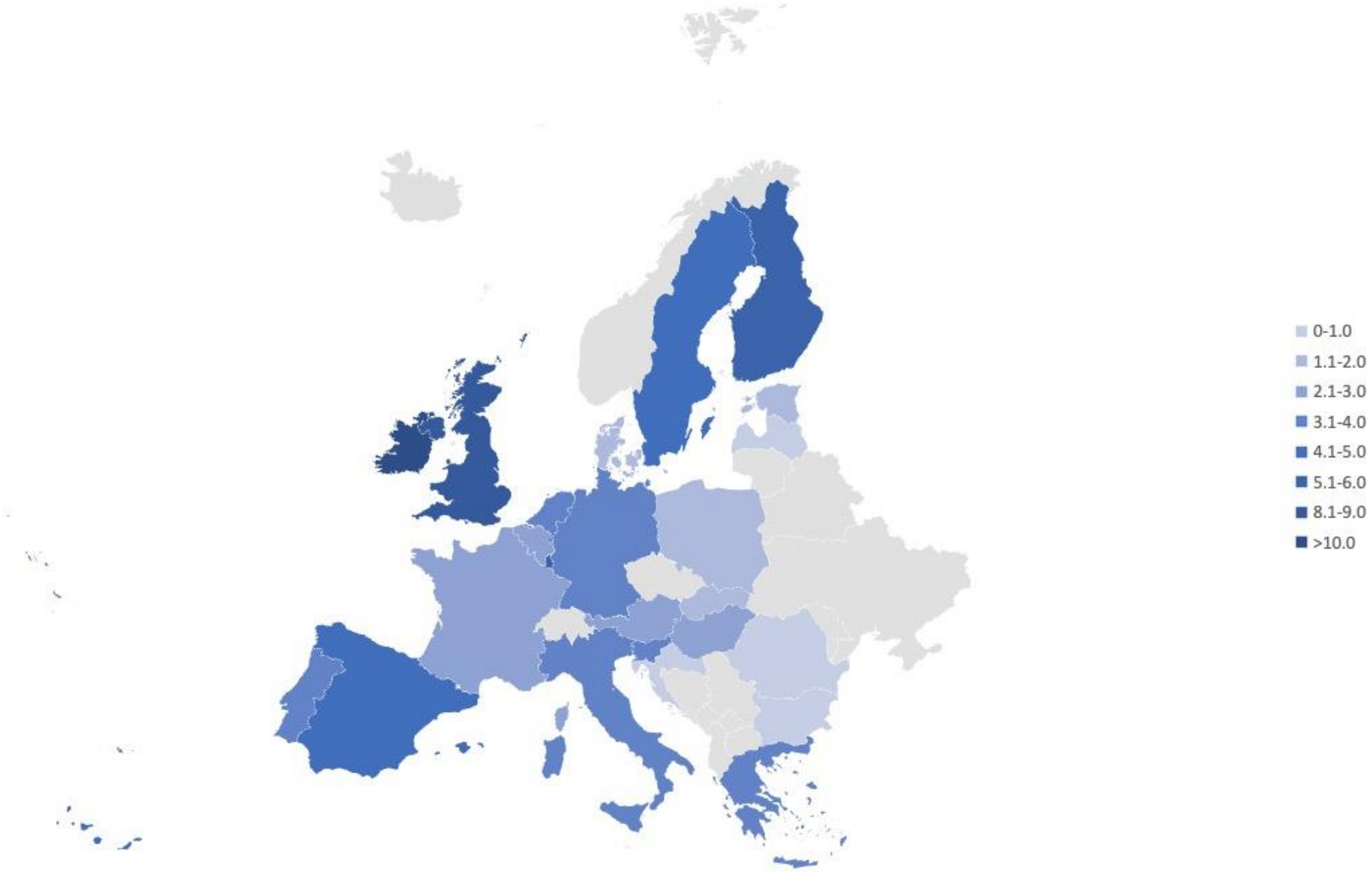

Supplementary Material S8 Broader analysis: comparison between narrow ("J84.1") and broader band diagnosis codeset ("J84")

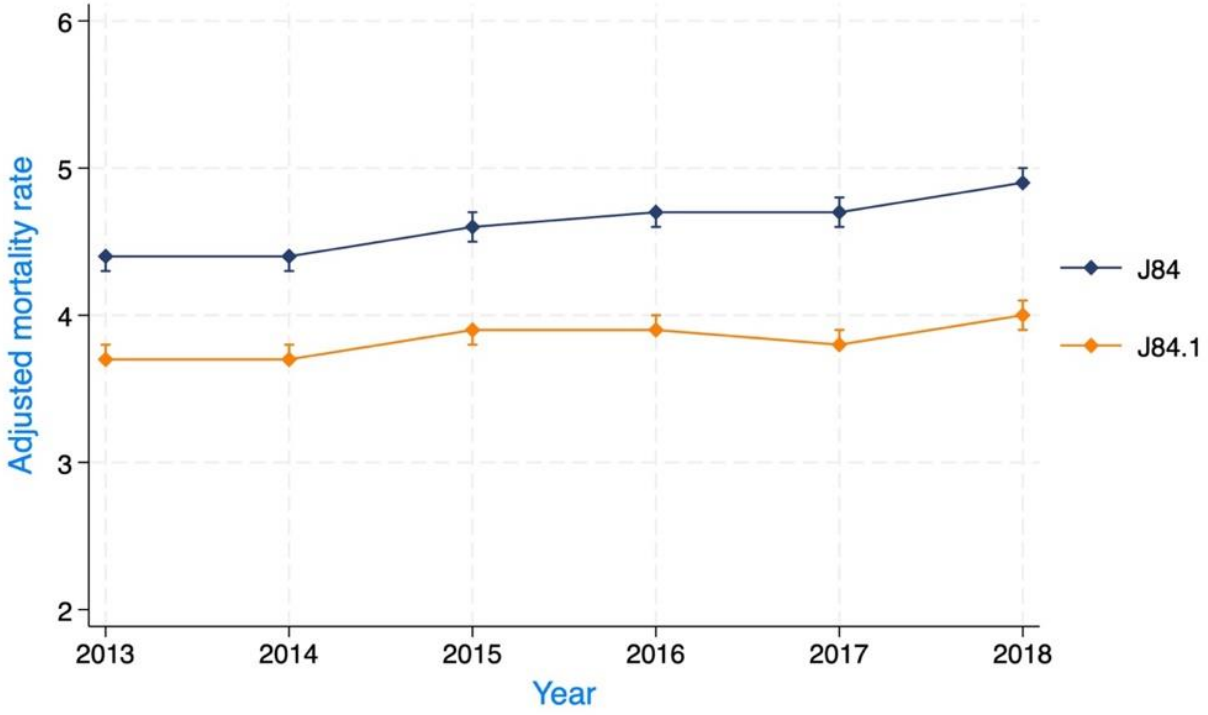

Supplementary Material S9: Broader analysis: crude mortality rates broader band diagnosis “J84” in 24 European Countries in 2013–2018

	Crude Mortality Rate per 100,000 Person-Years (95% CI) 2013			Crude Mortality Rate per 100,000 Person-Years (95% CI) **2014**			Crude Mortality Rate per 100,000 Person-Years (95% CI) **2015**			Crude Mortality Rate per 100,000 Person-Years (95% CI) **2016**			Crude Mortality Rate per 100,000 Person-Years (95% CI) **2017**			Crude Mortality Rate per 100,000 Person-Years (95% CI) **2018**			Overall Crude Mortality Rate per 100,000 Person-Years (95% CI) **2013-2018**		
Country	*Deaths*	*Crude rate*	*CI*	*Deaths*	*Crude rate*	*CI*	*Deaths*	*Crude rate*	*CI*	*Deaths*	*Crude rate*	*CI*	*Deaths*	*Crude rate*	*CI*	*Deaths*	*Crude rate*	*CI*	*Deaths*	*Crude rate*	*CI*
Austria	165	**1.92**	(1.68-2.27)	184	**2.16**	(1.84-2.50)	188	**2.27**	(1.97-2.62)	175	**2.08**	(1.79-2.42)	195	**3.35**	(2.90-3.86)	217	**2.46**	(2.14-2.81)	1124	**2.33**	(2.19-2.47)
Belgium	396	**3.55**	(3.22-3.92)	355	**3.17**	(2.86-3.52)	375	**3.34**	(3.02-3.69)	425	**3.76**	(3.41-4.13)	407	**3.70**	(3.35-4.08)	439	**5.89**	(5.53-6.47)	2397	**3.79**	(3.64-3.94)
Bulgaria	33	**0.76**	(0.55-1.07)	29	**0.72**	(0.50-1.04)	14	**0.24**	(0.14-0.40)	10	**0.29**	(0.14-0.54)	17	**0.28**	(0-16-0.45)	24	**0.59**	(0.38-0.87)	127	**0.46**	(0.38-0.54)
Croatia	20	**0.51**	(0.33-0.79)	21	**3.29**	(2.14-5.04)	22	**2.05**	(1.35-3.11)	23	**0.96**	(0.61-1.44)	24	**0.97**	(0.62-1.45)	32	**2.25**	(1.54-3.17)	142	**1.19**	(1.01-2.12)
Denmark	137	**2.44**	(2.07-2.89)	160	**2.84**	(2.43-3.32)	165	**3.01**	(2.58-3.51)	194	**3.40**	(2.94-3.91)	199	**5.59**	(4.84-6.43)	248	**4.43**	(3.90-5.02)	1103	**3.49**	(3.29-3.71)
Estonia	N/A	N/A	N/A	7	**1.21**	(0.57-2.53)	9	**13.07**	(6.80-25.12)	5	**9.25**	(3.00-21.59)	4	**4.88**	(1.33-12.48)	21	**3.05**	(1.89-4.66)	46	**3.12**	(2.28-4.16)
Finland	278	**7.63**	(6.78-8.58)	276	**7.68**	(6.82-8.64)	279	**7.56**	(6.72-8.50)	352	**9.48**	(8.51-10.52)	309	**8.70**	(7.76-9.73)	341	**9.08**	(8.14-10.01)	1835	**8.36**	(7.98-8.75)
France	1844	**2.81**	(2.87-2.94)	1946	**2.81**	(2.81-3.07)	2072	**3.12**	(2.99-3.25)	2108	**3.16**	(3.03-3.30)	2150	**3.22**	(3.08-3.36)	N/A	**N/A**	N/A	10120	**3.05**	(2.99-3.11)
Germany	2977	**3.70**	(3.57-3.83)	2997	**3.71**	(3.58-3.85)	3195	**3.93**	(3.80-4.07)	3359	**4.09**	(3.95-4.23)	3503	**4.24**	(4.11-4.39)	3526	**4.26**	(4.12-4.40)	19557	**3.99**	(3.94-4.05)
Greece	N/A	**N/A**	N/A	416	**3.81**	(3.46-4.19)	475	**4.37**	(4.00-4.79)	436	**4.19**	(3.80-4.60)	478	**4.44**	(4.05-4.86)	437	**4.07**	(3.70-4.47)	2242	**4.17**	(4.00-4.35)
Hungary	182	**1.84**	(1.59-2.12)	164	**1.66**	(1.42-1.93)	199	**2.02**	(1.76-2.32)	197	**2.00**	(1.73-8.67)	200	**2.04**	(1.77-2.34)	232	**2.44**	(2.14-2.78)	1174	**2.00**	(1.89-2.12)
Ireland	302	**6,55**	(5.83-7.33)	344	**7.42**	(6.67-8.24)	339	**11.75**	(10.57-13.08)	370	**7.83**	(7.05-8.67)	409	**14.43**	(13.07-15.90)	409	**13.58**	(12.30-14.97)	2173	**9.57**	(9.17-9.98)
Italy	2268	**3.80**	(3.65-3.96)	2278	**3.75**	(3.60-3.90)	2461	**4.05**	(3.89-4.21)	2426	**4.00**	(3.84-4.16)	2677	**4.42**	(4.25-4.59)	2836	**4.69**	(4.52-4.86)	14946	**4.12**	(4.05-4.18)
Luxembourg	11	**17.17**	(9.51-31.01)	13	**22.01**	(12.78-37.90)	10	**16.85**	(8.10-30.99)	12	**27.63**	(14.27-48.26)	12	**24.26**	(12.54-42.38)	15	**18.99**	(10.63-31.32)	73	**20.60**	(16.15-25.90)
Latvia	14	**1.38**	(0.82-2.33)	7	**5.69**	(2.71-11.94)	14	**4.06**	(2.22-6.81)	5	**0.69**	(0.22-1.60)	9	**3.24**	(1.48-6.15)	12	**0.78**	(0.40-1.37)	61	**1.52**	(1.16-1.95)
Netherlands	535	**3.19**	(2.93-3.47)	544	**3.23**	(2.97-3.52)	629	**3.72**	(3.44-4.02)	634	**3.87**	(3.57-4.18)	626	**3.66**	(3.38-3.96)	677	**3.94**	(3.65-4.25)	3645	**3.60**	(3.49-3.72)
Poland	396	**1.04**	(0.94-1.15)	418	**1.01**	(1.00-1.21)	439	**1.16**	(1.05-1.27)	466	**1.23**	(1.12-1.34)	515	**1.36**	(1.24-1.48)	470	**1.24**	(1.13-1.35)	2704	**1.19**	(1.14-1.23)
Portugal	348	**3.32**	(2.99-3.69)	402	**3.85**	(3.50-4.25)	421	**4.06**	(3.68-4.46)	469	**4.54**	(4.13-4.96)	408	**3.96**	(3.58-4.36)	403	**3.92**	(3.54-4.32)	2451	**3.94**	(3.78-4.10)
Romania	213	**1.06**	(0.93-1.22)	190	**0.95**	(0.83-1.10)	250	**1.30**	(1.14-1.47)	292	**1.48**	(1.31-1.66)	233	**1.19**	(1.04-1.35)	269	**1.38**	(1.22-1.55)	1447	**1.22**	(1.16-1.29)
Slovenia	45	**7.87**	(5.87-10.53)	54	**2.82**	(2.17-3.69)	52	**8.84**	(6.60-11.59)	59	**10.68**	(8.13-13.78)	57	**13.70**	(10.38-17.75)	75	**13.69**	(10.77-17.16)	342	**7.46**	(6.69-8.29)
Slovakia	78	**1.49**	(1.20-1.86)	91	**1.68**	(1.36-2.06)	84	**1.56**	(1.24-1.93)	97	**1.85**	(1.50-2.26)	74	**1.46**	(1.15-1.84)	99	**2.87**	(2.34-3.50)	523	**1.76**	(1.61-1.91)
Spain	2275	**4.87**	(4.67-5.07)	2362	**5.08**	(4.88-5.29)	2552	**5.49**	(5.28-5.71)	2627	**5.56**	(5.44-5.88)	2717	**5.84**	(5.62-6.06)	2648	**5.68**	(5.46-5.90)	15181	**5.43**	(5.35-5.52)
Sweden	423	**6.67**	(6.062-7.33)	442	**4.58**	(4.17-5.03)	519	**5.32**	(4.88-5.80)	515	**5.39**	(4.93-5.31)	534	**5.34**	(4.90-5.82)	539	**5.33**	(4.89-5.80)	2972	**5.36**	(5.17-5.56)
UK	5770	**9.02**	(8.78-9.26)	5977	**9.29**	(9.06-9.53)	6397	**9.86**	(9.62-10.11)	6655	**10.18**	(9.94-10.43)	6733	**10.23**	(9.98-10.47)	6966	**10.51**	(10.27-10.76)	38498	**9.86**	(9.76-9.95)
**All Europe**	**18710**	**4.01**	**(3.95-4.07)**	**19677**	**4.09**	**(4.38-4.15)**	**21160**	**4.41**	**(4.35-4.47)**	21911	**4.55**	**(4.49-4.61)**	**22490**	**4.70**	**(4.63-4.76)**	**20935**	**5.08**	**(5.01-5.15)**	124883	**4.46**	**(4.44-4.49)**

CI: confidence interval

Supplementary Material S10: Broader analysis: age- and sex- standardised mortality rates for IPF broad codeset in 24 European Countries in 2013–2018

	Standardized Mortality Rate per 100,000 Person-Years (95% CI) **2013**		Standardized Mortality Rate per 100,000 Person-Years (95% CI) **2014**		Standardized Mortality Rate per 100,000 Person-Years (95% CI) **2015**		Standardized Mortality Rate per 100,000 Person-Years (95% CI) **2016**		Standardized Mortality Rate per 100,000 Person-Years (95% CI) **2017**		Standardized Mortality Rate per 100,000 Person-Years (95% CI) **2018**		Overall Standardized Mortality Rate per 100,000 Person-Years (95% CI) **2013-2018**		AAPC	CI
Country	*Rate*	*IC*	*Rate*	*IC*	*Rate*	*IC*	*Rate*	*IC*	*Rate*	*IC*	*Rate*	*IC*	*Rate*	*IC*		
Austria	**2.20**	(1.90-2.60)	**2.40**	(2.10-2.80)	**2.40**	(2.10-2.80)	**2.20**	(1.90-2.50)	**2.30**	(2.00-2.70)	**2.60**	(2.20-2.90)	**2.40**	(2.20-2.50)	5.64	(-4.02-16.52)
Belgium	**3.90**	(3.60-4.30)	**3.50**	(3.10-3.90)	**3.60**	(3.20-4.00)	**4.00**	(3.60-4.40)	**3.80**	(3.40-4.20)	**4.00**	(3.60-4.40)	**3.80**	(3.70.4.00)	1.95	(-3.54-8.01)
Bulgaria	**0.50**	(0.30-0.70)	**0.40**	(0.30-0.60)	**0.20**	(0.10-0.30)	**0.10**	(0.10-0.20)	**0.20**	(0.10-0.40)	**0.40**	(0.20-0.50)	**0.50**	(0.40-0.60)	-7.86	(-33.42-20.99)
Croatia	**0.50**	(0.30-0.70)	**0.60**	(0.30-0.80)	**0.50**	(0.30-0.80)	**0.60**	(0.40-0.90)	**0.60**	(0.40-0.90)	**0.80**	(0.50-1.10)	**0.80**	(0.70-0.90)	8.34	(-4.55-25.61)
Denmark	**2.90**	(2.40-3.40)	**3.30**	(2.80-3.80)	**3.30**	(2.80-3.80)	**3.90**	(3.30-4.40)	**3.90**	(3.30-4.40)	**4.70**	(4.10-5.30)	**3.70**	(3.50-4.00)	12.94*	(3.92-23.25)
Estonia	**N/A**	N/A	**0.60**	(0.20-1.10)	**0.90**	(0.30-1.40)	**0.40**	(0.10-0.80)	**0.30**	(0.00-0.70)	**1.90**	(1.10-2.80)	**2.00**	(1.30-2.70)	1.54	(-0.82-4.11)
Finland	**5.70**	(5.10-6.40)	**5.50**	(4.90-6.20)	**5.50**	(4.90-6.20)	**6.80**	(6.10-7.50)	**5.80**	(5.60-6.40)	**6.20**	(5.60-6.90)	**6.00**	(5.70-6.30)	6.05	(-5.59-20.17)
France	**3.00**	(2.90-3.20)	**3.10**	(3.00-3.30)	**3.30**	(3.10-3.40)	**3.30**	(3.10-3.40)	**3.20**	(3.10-3.40)	**N/A**	N/A	**3.20**	(3.10-3.30)	1.90	(-2.31-6.22)
Germany	**3.60**	(3.50-3.80)	**3.60**	(3.50-3.70)	**3.70**	(3.60-3.80)	**3.80**	(3.70-3.90)	**3.90**	(3.80-4.00)	**3.90**	(3.70-4.00)	**3.80**	(3.70-3.80)	1.99*	(0.94-3.11)
Greece	**N/A**	N/A	**3.60**	(3.30-4.00)	**4.10**	(3.70-4.40)	**3.70**	(3.30-4.00)	**3.90**	(3.60-4.30)	**3.60**	(3.20-3.90)	**3.80**	(3.60-3.90)	-0.50	(-6.66-6.09)
Hungary	**2.20**	(1.90-2.50)	**2.00**	(1.70-2.30)	**2.40**	(2.10-2.70)	**2.30**	(2.00-2.70)	**2.40**	(2.00-2.70)	**2.70**	(2.30-3.00)	**2.30**	(2.20-2.50)	4.21	(-1.03-10.13)
Ireland	**11.30**	(10.00-12.60)	**12.60**	(11.20-13.90)	**11.90**	(10.60-13.20)	**12.40**	(11.10-13.60)	**13.50**	(12.20-14.80)	**13.00**	(11.70-14.30)	**12.50**	(12.00-13.10)	13.43	(-1.34-10.13)
Italy	**3.50**	(3.40-3.70)	**3.50**	(3.30-3.60)	**3.60**	(3.50-3.80)	**3.50**	(3.40-3.70)	**3.80**	(3.70-4.00)	**4.00**	(3.90-4.20)	**3.70**	(3.60-3.70)	2.91*	(0.83-5.15)
Luxembourg	**2.90**	(12.00-4.60)	**3.50**	(1.60-5.40)	**2.50**	(1.00-4.10)	**3.00**	(1.30-4.70)	**2.90**	(1.20-4.50)	**3.40**	(1.70-5.20)	**4.40**	(3.30-5.60)	1.49	(-8.99-13.49)
Latvia	**0.70**	(0.30-1.00)	**0.50**	(0.10-0.90)	**0.90**	(0.40-1.30)	**0.30**	(0.00-0.50)	**0.50**	(0.20-0.90)	**0.70**	(0.30-1.10)	**1.30**	(0.90-1.70)	-0.30	(-47.06-78.74)
Netherlands	**4.00**	(3.60-4.30)	**3.90**	(3.60-4.20)	**4.40**	(4.10-4.80)	**4.30**	(4.00-4.60)	**4.20**	(3.80-4.50)	**4.40**	(4.00-4.70)	**4.20**	(4.10-4.30)	2.07	(-1.77-6.28)
Poland	**1.40**	(1.20-1.50)	**1.40**	(1.30-1.50)	**1.40**	(1.30-1.50)	**1.50**	(1.40-1.70)	**1.70**	(1.50-1.80)	**1.50**	(1.30-1.60)	**1.50**	(.40-1.50)	2..61	(-0.01-5.31)
Portugal	**3.50**	(3.10-3.80)	**3.90**	(3.60-4.30)	**4.10**	(3.70-4.40)	**4.40**	(4.00-4.80)	**3.80**	(3.40-4.10)	**3.70**	(3.30-4.00)	**3.90**	(3.70-4.10)	0.54	(-6.42-8.12)
Romania	**1.30**	(1.10-1.50)	**1.10**	(1.00-1.30)	**1.50**	(1.30-1.70)	**1.70**	(1.50-1.90)	**1.40**	(1.20-1.60)	**1.60**	(1.40-1.80)	**1.40**	(1.40-1.50)	4.13	(-8.79-21.55)
Slovenia	**2.70**	(1.90-3.50)	**3.20**	(2.40-4.10)	**2.90**	(2.10-3.70)	**3.30**	(2.50-4.20)	**3.10**	(2.30-3.90)	**4.00**	(3.10-4.90)	**3.50**	(3.10-3.90)	11.00*	(0.17-23.60)
Slovakia	**2.10**	(1.60-2.50)	**2.40**	(1.90-2.90)	**1.90**	(1.50-2.30)	**2.50**	(2.00-3.00)	**1.90**	(1.40-2.30)	**2.30**	(1.80-2.70)	**2.30**	(2.10-2.50)	1.29	(-4.71-7.69)
Spain	**5.30**	(5.10-5.50)	**5.40**	(5.20-5.60)	**5.70**	(5.50-5.90)	**5.70**	(5.50-5.90)	**5.80**	(5.60-6.00)	**5.60**	(5.40-5.80)	**5.60**	(5.50-5.70)	1.54	(-0.83-4.12)
Sweden	**4.80**	(4.30-5.20)	**4.80**	(4.40-5.30)	**5.60**	(5.10-6.10)	**5.40**	(5.00-5.90)	**5.50**	(5.10-6.00)	**5.50**	(5.00-5.90)	**5.30**	(5.10-5.50)	2.94	(-1.20-7.45)
UK	**10.60**	(10.30-10.80)	**10.70**	(10.40-11.00)	**11.20**	(11.00-11.50)	**11.50**	(11.20-11.70)	**11.40**	(11.10-11.60)	**11.50**	(11.20-11.70)	**11.10**	(11.00-11.30)	1.93*	(0.98-2.88)
**All Europe**	**4.40**	**(4.30-4.40)**	**4.40**	**(4.30-4.40)**	**4.60**	**(4.50-4.70)**	**4.70**	**(4.60-4.70)**	**4.70**	**(4.60-4.80)**	**4.90**	**(4.90-5.00)**	**4.60**	**(4.60-4.60)**	**3.02***	**(2.17-3.91)**

AAPC: average annual percent change; CI: confidence interval; *: statistically significant.

Supplementary Material S11: Broader analysis: overall row deaths, age- and sex- standardised mortality rates for IPF broad codeset ("J84") in Europe in 2013–2018, age- and sex- stratum data

	Standardised Mortality Rate per 100,000 Person-Years (95% CI) **2013**			Standardised Mortality Rate per 100,000 Person-Years (95% CI) **2014**			Standardised Mortality Rate per 100,000 Person-Years (95% CI) **2015**			Standardised Mortality Rate per 100,000 Person-Years (95% CI) **2016**		
	Row deaths	*Rate*	*IC*	Row deaths	*Rate*	*IC*	Row deaths	*Rate*	*IC*	Row deaths	*Rate*	*IC*
Total population	16148	**4.40**	(4.30-4.40)	16877	**4.40**	(4.30-4.40)	17885	**4.60**	(4.50-4.70)	18331	**4.70**	(4.60-4.70)
Sex												
Female	6484	**3.40**	(3.30-3.50)	6662	**3.40**	(3.30-3.50)	7060	**3.60**	(3.50-3.70)	7220	**3.70**	(3.70-3.80)
Male	9664	**5.30**	(5.20-5.40)	10215	**5.30**	(5.20-5.40)	10825	**5.60**	(5.50-5.70)	11111	**5.60**	(5.50-5.70)
Age group (years)												
0-54	386	**0.20**	(0.10-0.20)	332	**0.10**	(0.10-0.10)	339	**0.20**	(0.10-0.20)	382	**0.20**	(0.20-0.20)
55-59	352	**1.50**	(1.30-1.60)	340	**1.40**	(1.30-1.50)	387	**1.60**	(1.40-1.70)	359	**1.50**	(1.30-1.60)
60-64	759	**3.20**	(3.00-3.40)	752	**3.20**	(3.00-3.40)	739	**3.20**	(3.00-3.40)	791	**3.30**	(3.10-3.50)
65-69	1268	**6.20**	(5.90-6.50)	1411	**6.60**	(6.30-6.90)	1401	**6.40**	(6.10-6.70)	1472	**6.60**	(6.30-6.90)
70-74	2214	**12.30**	(11.80-12.80)	2336	**13.00**	(12.60-13.50)	2223	**12.50**	(12.00-13.00)	2257	**13.00**	(12.50-13.50)
75-79	3270	**21.50**	(20.90-22.20)	3494	**21.90**	(21.20-22.60)	3710	**23.40**	(22.70-24.00)	3767	**23.30**	(22.60-24.00)
80-84	3595	**32.40**	(31.40-33.40)	3724	**32.00**	(31.00-33.00)	4119	**35.60**	(34.60-36.60)	4162	**36.20**	(35.20-37.20)
> 85	4304	**44.80**	(43.60-46.10)	4488	**43.60**	(42.40-44.80)	4967	**47.00**	(45.80-48.20)	5141	**47.90**	(46.70-49.10)

→ continue

→ continue

	Standardised Mortality Rate per 100,000 Person-Years (95% CI) **2017**			Standardised Mortality Rate per 100,000 Person-Years (95% CI) **2018**			Overall Standardised Mortality Rate per 100,000 Person-Years (95% CI) **2013-2018**			AAPC	CI
	Row deaths	*Rate*	*IC*	Row deaths	*Rate*	*IC*	Row deaths	*Rate*	*IC*		
Total population	18500	**4.70**	(4.60-4.80)	17026	**4.90**	(4.90-5.00)	104767	**4.60**	(4.60-4.60)	3.02*	2.17-3.91)
Sex											
Female	7139	**3.70**	(3.60-3.70)	6418	**3.80**	(3.80-3.90)	40983	**3.60**	(3.60-3.60)	2.81*	(1.56-4.11)
Male	11361	**5.70**	(5.60-5.80)	10608	**6.10**	(6.00-6.20)	63784	**5.60**	(5.60-5.70)	2.69*	(1.28-4.18)
Age group (years)											
0-54	320	**0.10**	(0.10-0.20)	319	**0.20**	(0.20-0.20)	2078	**0.20**	(0.20-0.20)	2.32	(-3.39-8.37)
55-59	345	**1.50**	(1.40-1.70)	294	**1.40**	(1.30-1.60)	2077	**1.50**	(1.40-1.50)	0.32	(-4.56-5.51)
60-64	753	**3.30**	(3.10-3.50)	687	**3.30**	(3.10-3.50)	4481	**3.20**	(3.20-3.30)	1.27	(-0.18-2.74)
65-69	1406	**6.20**	(5.90-6.40)	1253	**6.50**	(6.20-6.80)	8211	**6.40**	(6.30-6.50)	0.06	(-2.23-2.47)
70-74	2186	**12.40**	(12.00-12.90)	2310	**14.30**	(13.80-14.80)	13526	**12.90**	(12.70-13.10)	1.98	(-1.74-5.86)
75-79	3718	**23.40**	(22.70-24.10)	3374	**24.40**	(23.70-25.20)	21333	**23.00**	(22.70-23.30)	2.36*	(1.18-3.55)
80-84	4267	**36.30**	(35.40-37.30)	3889	**37.70**	(36.60-38.80)	23756	**35.10**	(34.60-35.50)	3.31*	(1.47-5.29)
> 85	5505	**50.20**	(49.00-51.40)	4900	**52.10**	(50.80-53.40)	29305	**47.70**	(47.20.48.20)	3.50*	(2.45-4.60)

AAPC: average annual percent change; CI: confidence interval; *: statistically significant

Supplementary Material S12: crude and age- and sex- standardised IPF-CS mortality rates in 24 European Countries in 2019-2020

	Narrow codeset band										Broad codeset band									
	2019					2020					2019					2020				
	Crude Mortality Rate per 100,000 Person-Years (95% CI)			Standardized Mortality Rate per 100,000 Person-Years (95% CI)		Crude Mortality Rate per 100,000 Person-Years (95% CI)			Standardized Mortality Rate per 100,000 Person-Years (95% CI)		Crude Mortality Rate per 100,000 Person-Years (95% CI)			Standardized Mortality Rate per 100,000 Person-Years (95% CI)		Crude Mortality Rate per 100,000 Person-Years (95% CI)			Standardized Mortality Rate per 100,000 Person-Years (95% CI)	
Country	*Deaths*	*Crude rate*	*IC*	*Rate*	*IC*	*Deaths*	*Crude rate*	*IC*	*Rate*	*IC*	*Deaths*	*Crude rate*	*IC*	*Rate*	*IC*	*Deaths*	*Crude rate*	*IC*	*Rate*	*IC*
Austria	166	**1.95**	(1.67-2.27)	**2.00**	(1.70-2.30)	100	**1.17**	(0.96-1.42)	**1.10**	(0.90-1.40)	192	**2.25**	(1.96-2.60)	**2.30**	(1.90-2.60)	125	**1.46**	(1.23-1.74)	**1.40**	(1.20-1.70)
Belgium	389	**3.40**	(3.07-3.75)	**3.50**	(3.10-3.80)	313	**4.13**	(3.70-4.62)	**2.70**	(2.40-3.00)	475	**4.15**	(3.79-4.54)	**4.30**	(3.90-4.70)	424	**5.60**	(5.09-6.16)	**3.70**	(3.30-4.10)
Bulgaria	53	**0.76**	(0.58-0.99)	**0.80**	(0.50-1.00)	40	**0.93**	(0.68-1.72)	**0.60**	(0.40-0.80)	55	**0.79**	(0.60-1.02)	**0.80**	(0.60-1.00)	48	**1.12**	(0.84-1.49)	**0.70**	(0.50-0.90)
Croatia	19	**1.67**	(1.07-2.62)	**0.50**	(0.30-0.70)	13	**0.41**	(0.24-0.70)	**0.30**	(0.10-0.50)	24	**2.11**	(1.42-3.15)	**0.60**	(0.40-0.80)	21	**0.66**	(0.43-1.01)	**0.50**	(0.30-0.70)
Denmark	128	**3.31**	(2.79-3.94)	**2.40**	(2.00-2.80)	134	**3.79**	(3.20-4.49)	**2.40**	(2.00-2.80)	239	**6.18**	(5.45-7.02)	**4.40**	(3.90-5.00)	234	**6.62**	(5.83-7.53)	**4.20**	(3.70-4.80)
Estonia	15	**2.63**	(1.58-4.35)	**1.30**	(0.60-2.00)	20	**2.89**	(1.87-4.48)	**1.70**	(0.90-2.40)	18	**13.04**	(8.21-20.69)	**1.60**	(0.90-2.40)	27	**10.41**	(7.14-15.18)	**2.20**	(1.40-3.10)
Finland	339	**4.04**	(3.87-4.23)	**6.10**	(5.40-6.70)	264	**4.94**	(4.38-5.58)	**4.50**	(4.00-5.10)	382	**10.13**	(9.17-11.20)	**6.90**	(6.20-7.60)	306	**8.49**	(7.59-9.50)	**5.30**	(4.70-5.90)
France	N/A	**N/A**	N/A	**N/A**	N/A	1760	**2.61**	(2.50-2.74)	**2.50**	(2.40-2.60)	N/A	**N/A**	N/A	**N/A**	N/A	2098	**3.12**	(2.99-3.25)	**3.00**	(2.90-3.10)
Germany	3334	**4.02**	(3.88-4.16)	**3.60**	(3.40-3.70)	3170	**3.81**	3.68-3.95)	**3.30**	(3.20-3.40)	3735	**4.50**	(4.36-4..64)	**4.00**	(3.90-4.10)	3625	**4.36**	(4.22-4.50)	**3.80**	(3.70-3.90)
Greece	440	**4.25**	(3.87-4.67)	**3.60**	(3.20-3.90)	372	**3.47**	(3.14-3.84)	**3.00**	(2.60-3.30)	506	**4.89**	(4.85-5.34)	**4.10**	(3.70-4.50)	424	**3.96**	(3.60-4.35)	**3.40**	(3.10-3.70)
Hungary	192	**1.96**	(1.71-2.26)	**2.10**	(1.80-2.50)	170	**1.74**	(1.50-2.02)	**1.90**	(1.60-2.20)	215	**2.20**	(1.93-2.51)	**2.40**	(2.10-2.70)	189	**1.93**	(1.68-2.23)	**2.20**	(1.80-2.50)
Ireland	408	**8.57**	(7.78-9.44)	**12.60**	(11.40-13.80)	308	**9.89**	(8.84-11.06)	**9.10**	(8.00-10.10)	471	**9.89**	(9.04-10.83)	**14.50**	(13.20-15.80)	378	**12.14**	(10.97-13.42)	**0.10**	(9.90-12.20)
Italy	2605	**4.35**	(4.19-4.52)	**3.60**	(3.50-3.70)	2238	**3.75**	(3.60-3.91)	**3.00**	(2.90-3.20)	2920	**4.88**	(4.71-5.06)	**4.10**	(3.90-4.20)	6180	**10.36**	(10.11-10.62)	**8.40**	(8.20-8.60)
Luxembourg	17	**5.88**	(3.66-9.46)	**3.70**	(1.90-5.50)	23	**4.12**	(2.74-6.20)	**4.50**	(2.60-6.30)	14	**0.40**	(12.08-34.45)	**3.30**	(1.60-5.00)	19	**8.93**	(12.08-29.68)	**4.10**	(2.30-6.00)
Latvia	14	**1.44**	(0.85-2.43)	**0.80**	(0.40-1.20)	10	**3.94**	(2.12-7.32)	**0.60**	(0.20-1.00)	14	**3.99**	(2.36-6.74)	**0.80**	.40-1.30)	12	**4.73**	(2.68-8.32)	**0.70**	(0.30-1.10)
Netherlands	566	**3,28**	(3.02-3.56)	**3.50**	(3.20-3.80)	487	**3.01**	(2.76-3.29)	**2.90**	(2.70-3.20)	728	**4.21**	(3.92-4.53)	**4.60**	(4.20-4.90)	610	**3.76**	(3.49-4.09)	**3.70**	(3.40-4.00)
Poland	384	**1.01**	(0.92-1.12)	**1.20**	(1.10-1.30)	397	**1.04**	(0.95-1.15)	**1.20**	(1.10-1.40)	587	**1.55**	(1.43-1.68)	**1.80**	(.170-2.00)	600	**1.58**	(1.46-1.71)	**1.90**	(1.70-2.00)
Portugal	325	**3.16**	(2.84-3.53)	**2.90**	(2.60-3.20)	292	**2.83**	(2.53-3.18)	**2.50**	(2.20-2.80)	428	**4.16**	(3.79-4.58)	**3.80**	(3.50-4.20)	365	**3.54**	(3.20-3.93)	**3.20**	(2.90-3.50)
Romania	129	**0.66**	(0.56-0.79)	**0.70**	(0.60-0.90)	136	**0.70**	(0.60-0.83)	**0.80**	(0.60-0.90)	265	**1.37**	(1.21-1.54)	**1.50**	(1.30-1.70)	439	**2.71**	(2.07-2.49)	**2.50**	(2.30-2.70)
Slovenia	74	**5.42**	(4.31-6.80)	**3.70**	(2.80-4.50)	43	**3.50**	(2.60-4.73)	**2.10**	(1.50-2.80)	79	**11.16**	(8.95-13.91)	**4.00**	(3.10-4.80)	43	**7.51**	(5.58-10.14)	**2.20**	(1.50-2.80)
Slovakia	55	**1.04**	(0.80-1.36)	**1.30**	(1.00--1.70)	53	**1.04**	(0.80-1.36)	**1.20**	(0.90-1.60)	90	**1.71**	(1.39.2.10)	**2.10**	(1.60-2.50)	88	**1.73**	(1.40-2.13)	**2.10**	(1.60-2.50)
Spain	1898	**8.99**	(8.08-10.00)	**3.90**	(3.70-4.10)	1689	**3.57**	(3.40-3.74)	**3.40**	(3.30-3.60)	2542	**5.42**	(5.21-5.63)	**5.20**	(5.00-5.40)	2367	**5.00**	(4.80-5.21)	**4.80**	(4.60-5.00)
Sweden	560	**5.47**	(5.04-5.95)	**5.50**	(5.10-6.00)	478	**4.63**	(4.23-5.06)	**4.60**	(4.20-5.00)	622	**6.08**	(6.20-6.58)	**6.20**	(5.70-6.70)	520	**5.03**	(4.62-5.49)	**5.00**	(4.60-5.50)
UK	N/A	**N/A**	N/A	**N/A**	N/A	N/A	**N/A**	N/A	**N/A**	N/A	N/A	**N/A**	N/A	**N/A**	N/A	N/A	**N/A**	N/A	**N/A**	N/A
**All Europe**	**14129**	3.42	**(3.36-3.48)**	3.30	**(3.20-3.30)**	**14530**	3.01	**(2.96-3.06)**	2.80	**(2.80-2.80)**	**16620**	4.15	**(4.08-4.22)**	3.90	**(3.90-4.00)**	**21162**	4.65	**(4.58-4.71)**	4.2	**(4.20-4.40)**

IC: confidence interval

Supplementary Material S13: annual point prevalence rate for each European Country along with 95% CI in 2013-2018 (GDB extracted data). Prevalence rates are reported per 100,000 people.

Country	*2013*			*2014*			*2015*			*2016*			*2017*			*2018*		
	Prevalence rate	*95% CI*		*Prevalence rate*	*95% CI*		*Prevalence rate*	*95% CI*		*Prevalence rate*	*95% CI*		*Prevalence rate*	*95% CI*		*Prevalence rate*	*95% CI*	
		UCI	*LCI*		*UCI*	*LCI*		*UCI*	*LCI*		*UCI*	*LCI*		*UCI*	*LCI*		*UCI*	*LCI*
Austria	**94.40**	107.72	83.07	**92.68**	105.73	81.57	**92.33**	105.35	81.14	**96.51**	109.62	84.20	**100.71**	114.97	86.62	**97.05**	111.37	84.06
Belgium	**91.06**	101.51	80.39	**90.26**	100.96	79.74	**90.37**	101.31	79.99	**92.86**	104.19	81.40	**95.37**	108.02	82.31	**92.99**	104.98	81.00
Bulgaria	**57.91**	70.48	47.19	**58.60**	71.19	47.89	**59.45**	72.40	48.64	**63.12**	76.88	51.44	**66.79**	81.48	54.20	**67.62**	83.02	54.79
Croatia	**86.07**	100.01	73.46	**85.20**	99.05	72.29	**85.03**	99.22	72.06	**90.43**	105.85	76.01	**95.94**	113.17	79.76	**94.46**	111.59	78.47
Denmark	**82.44**	90.63	74.45	**82.05**	90.42	73.87	**82.05**	90.87	73.43	**82.00**	91.44	72.88	**82.26**	92.78	72.65	**83.33**	93.33	73.52
Estonia	**65.07**	76.12	55.43	**65.52**	76.91	55.74	**66.08**	77.89	56.16	**67.57**	79.43	56.92	**68.98**	81.94	57.85	**69.46**	82.27	58.12
Finland	**119.09**	132.57	105.80	**119.73**	133.74	106.41	**121.11**	135.83	107.18	**124.61**	140.10	109.49	**127.46**	145.07	110.70	**125.66**	142.14	109.38
France	**71.63**	80.20	63.59	**72.47**	81.36	64.29	**73.33**	82.79	65.01	**74.31**	83.98	65.37	**75.33**	85.86	65.54	**76.28**	86.86	66.35
Germany	**99.15**	109.93	88.43	**100.61**	111.42	89.50	**101.72**	112.94	90.18	**103.67**	115.43	92.25	**105.03**	117.98	92.99	**101.15**	114.32	88.99
Greece	**68.09**	76.52	59.79	**70.63**	79.26	61.96	**73.06**	82.51	63.96	**75.63**	85.49	65.76	**77.41**	88.83	66.77	**78.03**	89.36	67.26
Hungary	**81.04**	93.31	70.74	**82.19**	94.70	71.66	**83.52**	96.16	73.11	**86.04**	99.26	74.55	**88.55**	102.80	76.47	**89.87**	104.29	77.60
Ireland	**108.86**	120.98	97.19	**112.40**	125.08	99.90	**115.21**	128.82	101.75	**115.21**	128.95	100.98	**115.31**	131.43	100.11	**118.40**	134.37	102.59
Italy	**129.36**	147.55	111.79	**128.14**	146.34	110.25	**128.44**	147.20	110.21	**132.65**	152.71	112.82	**137.29**	158.99	116.06	**136.87**	159.56	115.16
Latvia	**81.80**	95.52	69.53	**81.77**	95.70	69.42	**82.13**	96.51	69.28	**86.52**	102.21	73.01	**90.69**	108.63	75.68	**85.89**	102.84	71.37
Luxembourg	**68.45**	76.27	60.76	**68.45**	76.23	60.56	**68.81**	76.80	60.84	**69.83**	78.41	61.23	**70.76**	80.07	61.54	**69.44**	79.11	60.34
Netherlands	**80.03**	90.73	70.05	**81.82**	92.73	71.60	**83.54**	95.04	72.93	**85.16**	97.18	73.85	**86.94**	100.43	74.83	**88.56**	101.79	76.13
Poland	**71.67**	81.24	62.29	**73.28**	82.83	64.22	**74.83**	84.43	65.75	**76.93**	86.97	67.50	**78.78**	89.31	68.79	**79.66**	90.28	69.36
Portugal	**92.36**	101.93	83.28	**94.35**	104.30	84.89	**95.97**	106.41	86.13	**95.65**	106.12	85.34	**95.22**	106.55	84.03	**96.74**	108.83	85.14
Romania	**91.62**	103.97	80.26	**92.48**	105.99	80.91	**93.44**	107.28	81.62	**95.04**	109.74	82.67	**96.81**	111.49	83.36	**98.32**	113.40	84.35
Slovakia	**67.99**	79.55	58.42	**69.67**	81.51	59.87	**71.51**	83.79	61.28	**74.46**	87.03	63.53	**77.18**	90.96	65.22	**78.55**	92.36	66.50
Slovenia	**101.15**	115.09	87.50	**101.39**	115.46	87.63	**102.13**	116.59	88.03	**106.49**	122.17	91.59	**111.00**	128.96	94.92	**109.03**	127.16	92.81
Spain	**134.60**	147.91	121.14	**136.33**	150.29	122.37	**137.69**	151.76	123.45	**138.10**	151.96	123.16	**138.45**	153.86	122.60	**140.84**	155.83	125.22
Sweden	**109.50**	130.49	90.29	**110.23**	131.33	91.12	**111.28**	132.78	91.60	**114.76**	138.50	93.57	**118.01**	144.54	95.17	**117.08**	143.48	93.80
United Kingdom	**139.06**	158.33	121.02	**141.25**	161.41	122.40	**143.16**	164.07	123.68	**144.44**	166.80	123.99	**146.10**	170.21	124.17	**148.85**	174.38	125.46
**All Europe**	**91.35**	103.69	79.83	**92.15**	104.75	80.42	**93.17**	106.20	81.14	95.50	109.18	82.65	**97.77**	112.85	83.85	**97.67**	112.79	83.66

CI: confidence interval: LCI: lower CI; UCI: upper CI; GDB: global burden of disease

Supplementary Material S14: overall ILDs and sarcoidosis prevalence rates in 2013-2018. The Y-axis reports prevalence rates indicated as number of cases per 100,000 people.

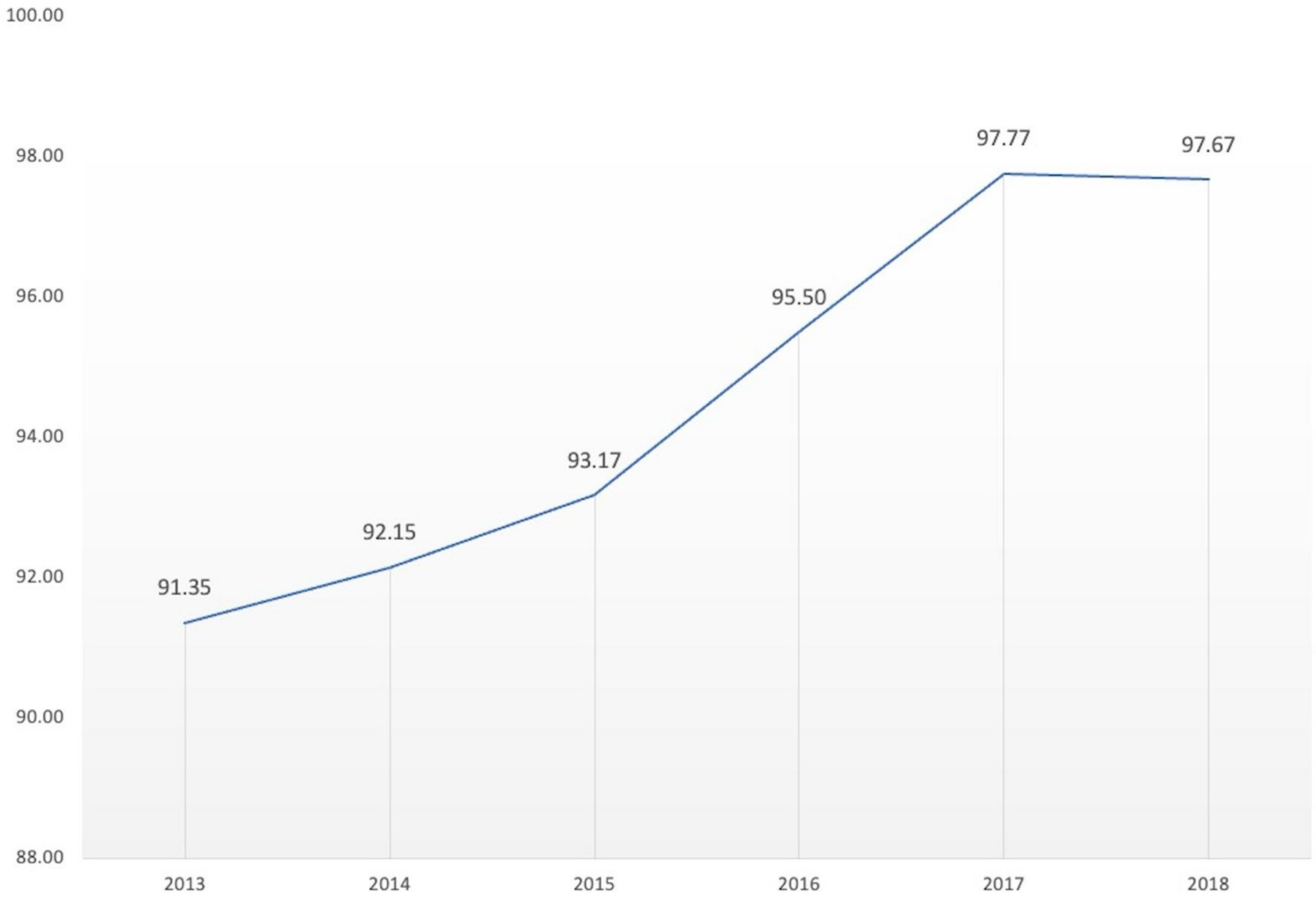

Supplementary Material S15: average prevalence rates along with 95% CI and relative belonging quartiles of ILDs and sarcoidosis from 2013-2018 in 24 European Countries (Global Burden of Disease data)

Country	Prevalence rate (per 100,000)	95% CI		Quartile
		LCI	*UCI*	
Austria	95.61	92.33	100.71	3
Belgium	92.15	90.26	95.37	3
Bulgaria	62.25	57.91	67.62	1
Croatia	89.52	85.03	95.94	2
Denmark	82.35	82.00	83.33	2
Estonia	67.11	65.07	69.46	1
Finland	122.94	119.09	127.46	4
France	73.89	71.63	76.28	1
Germany	101.89	99.15	105.03	3
Greece	73.81	68.09	78.03	1
Hungary	85.20	81.04	89.87	2
Ireland	114.23	108.86	118.40	4
Italy	132.12	128.14	137.29	4
Latvia	84.80	81.77	90.69	2
Luxembourg	69.29	68.45	70.76	1
Netherlands	84.34	80.03	88.56	2
Poland	75.86	71.67	79.66	2
Portugal	95.05	92.36	96.74	3
Romania	94.62	91.62	98.32	3
Slovenia	73.23	67.99	78.55	3
Slovakia	105.20	101.15	111.00	1
Spain	137.67	134.60	140.84	4
Sweden	113.47	109.50	118.01	4
UK	143.81	139.06	148.85	4

CI: confidence interval

Supplementary Material S16: Average prevalence rates of ILDs and sarcoidosis in 24 European Countries in 2013-2018 (Global Burden of Disease data). Prevalence rates are displayed according to the quartile of belonging.

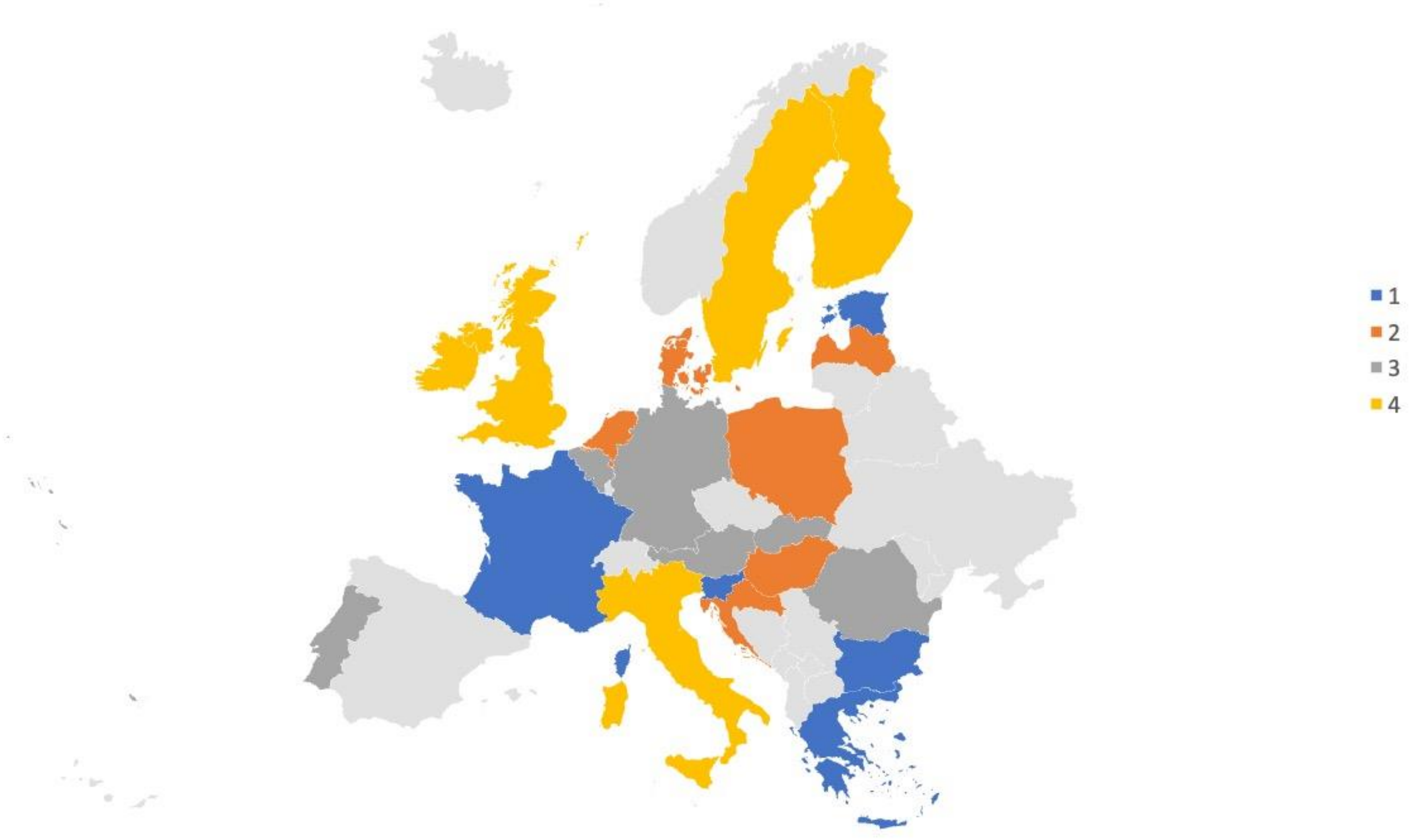

**Supplementary Material S17**: annual prevalence rate change for each European Country and overall, along with 95% CI from 2010-2019 (GDB extracted data).			
**Country**	**Annual prevalence rate change**	**95% CI**	
		LCI	*UCI*
Austria	-0.12*	-0.18	-0.05
Belgium	-0.08	-0.16	0.01
Bulgaria	0.21	0.14	0.29
Croatia	0.01	-0.08	0.10
Denmark	0.03	-0.04	0.09
Estonia	0.10*	0.03	0.17
Finland	0.01	-0.06	0.08
France	0.12	0.05	0.19
Germany	-0.02	-0.09	0.05
Greece	0.30*	0.21	0.40
Hungary	0.17*	0.10	0.23
Ireland	0.25*	0.16	0.34
Italy	0.00	-0.07	0.08
Latvia	-0.10*	-0.17	-0.01
Luxembourg	-0.05	-0.12	0.02
Netherlands	0.21*	0.13	0.29
Poland	0.18*	0.12	0.25
Portugal	0.15*	0.09	0.21
Romania	0.12*	0.05	0.19
Slovakia	0.25*	0.17	0.33
Slovenia	0.02	-0.07	0.10
Spain	0.12*	0.05	0.18
Sweden	0.04	-0.03	0.13
United Kingdom	0.15*	0.08	0.23
**All Europe**	**0.08**	**0.01**	**0.16**
CI: confidence interval: LCI: lower CI; UCI: upper CI; GDB: global burden of disease			
