# Supplementary material for: Mortality trends in idiopathic pulmonary fibrosis in Europe between 2013 and 2018
Source: Eur Respir J. 2024 Aug 22;64(2):2302080. doi: 10.1183/13993003.02080-2023 (PMC11339406; doi:10.1183/13993003.02080-2023)
Supplement: Supplementary file 2 [file ERJ-02080-2023.Shareable.pdf]

EUROPEAN RESPIRATORY JOURNAL
ORIGINAL RESEARCH ARTICLE
F. GONNELLI ET AL.

SHAREABLE PDF

Mortality trends in idiopathic pulmonary fibrosis in Europe between 2013 and 2018

Francesca Gonnelli, Martina Bonifazi and Richard Hubbard

Main objective

To provide an estimate of IPF-related mortality rates and trends for 24 EU countries in 2013–2018

Methods

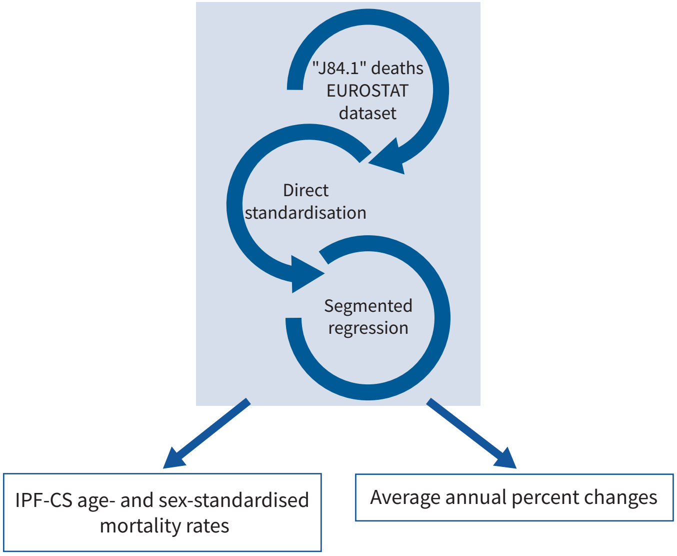

Results

↑ IPF-CS mortality rate in Europe, >17 000 deaths per year

High probability of under-reporting

Marked geographical differences among the EU countries

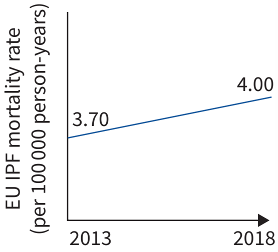

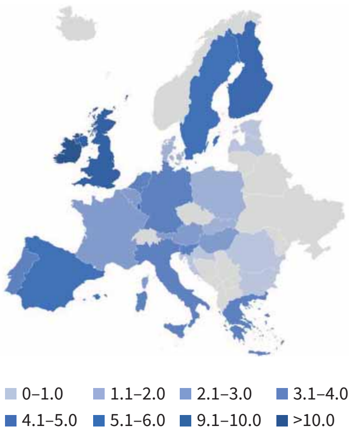

Overall EU mean mortality rate:
3.90 IPF-CS deaths per 100 000 person-years

GRAPHICAL ABSTRACT Overview of the study. IPF: idiopathic pulmonary fibrosis; IPF-CS: idiopathic pulmonary fibrosis-clinical syndrome; EU: European Union.

EUROPEAN RESPIRATORY JOURNAL
ORIGINAL RESEARCH ARTICLE
F. GONNELLI ET AL.

SHAREABLE PDF

Mortality trends in idiopathic pulmonary fibrosis in Europe between 2013 and 2018

Francesca Gonnelli [1,2], Martina Bonifazi [2,3] and Richard Hubbard [1]

[1]Lifespan and Population Health, University of Nottingham, Nottingham, UK. [2]Respiratory Unit, Department of Biomedical Sciences and Public Health, Polytechnic University of Marche, Ancona, Italy. [3]Interstitial Lung Diseases, Pleural Diseases and Bronchiectasis Unit, Azienda Ospedaliero-Universitaria delle Marche, Ancona, Italy.

Corresponding author: Francesca Gonnelli (Francesca.Gonnelli@nottingham.ac.uk)

Check for updates

Shareable abstract (@ERSpublications)
The mortality rate of idiopathic pulmonary fibrosis is on the rise across Europe. Considerable differences exist among countries. Currently, more than 17 000 deaths from IPF are recorded each year in Europe, with a high probability of underestimation. https://bit.ly/3yLyujQ

Cite this article as: Gonnelli F, Bonifazi M, Hubbard R. Mortality trends in idiopathic pulmonary fibrosis in Europe between 2013 and 2018. *Eur Respir J* 2024; 64: 2302080 [DOI: 10.1183/13993003.02080-2023].

This extracted version can be shared freely online.

Copyright ©The authors 2024.

This version is distributed under the terms of the Creative Commons Attribution Licence 4.0.

This article has an editorial commentary: https://doi.org/10.1183/13993003.01305-2024

Received: 21 Nov 2023
Accepted: 25 May 2024

Abstract

Background Previous research has suggested that the incidence of idiopathic pulmonary fibrosis (IPF) is increasing in the UK and elsewhere. The aim of this study is to provide contemporary estimates of IPF mortality rates across 24 European Union (EU) countries from 2013 to 2018, using death certificate data from the European Statistics Institution (EUROSTAT) database.

Methods We extracted country data for IPF (International Classification of Diseases, 10th Revision: code J84.1) mortality from the EUROSTAT dataset. We calculated country-, age- and sex-specific death registration rates between 2013 and 2018. We used direct standardisation to compare rates between countries. We calculated annual trends in mortality rate ratios using a segmented regression model.

Results The overall standardised mortality rate in 24 EU countries during this period was 3.90 (95% CI 3.80–3.90) per 100 000 person-years, with the rate rising from 3.70 in 2013 to 4.00 in 2018 (average annual percent change 1.74%, 95% CI 0.91–2.59%). We observed substantial inter-country differences, with the highest rates detected in Ireland, the UK and Finland, the lowest rate in Bulgaria, and middle rates in Germany, Greece, Italy, the Netherlands, Portugal and Slovenia.

Conclusions The IPF mortality rate is increasing across Europe. There are currently more than 17 000 deaths recorded from IPF each year in Europe but the marked geographical differences we observed suggest that this figure may underestimate the true rate considerably.
